# Microplastics as Vectors Influencing Oxidative Stress, Inflammation, and Endocrine Function During Early Development

**DOI:** 10.3390/ijms27125452

**Published:** 2026-06-16

**Authors:** Natalia Kurhaluk, Renata Kołodziejska, Anna Rymuszka, Rafał Bilski, Karolina Kaczorowska-Bilska, Vladimir Tomin, Piotr Kamiński, Halina Tkaczenko

**Affiliations:** 1 Institute of Biology, Pomeranian University in Słupsk, Arciszewski St. 22B, 76-200 Słupsk, Poland; 2 Department of Medical Biology and Biochemistry, Collegium Medicum in Bydgoszcz, Nicolaus Copernicus University in Toruń, M. Karłowicz St. 24, 85-092 Bydgoszcz, Poland; renatak@cm.umk.pl (R.K.);; 3 Department of Physiology and Toxicology, Faculty of Medicine, The John Paul II Catholic University of Lublin, Konstantynów St. 1I, 20-708 Lublin, Poland; anna.rymuszka@kul.pl; 4 Clinic of Hematology, Ludwik Rydygier Collegium Medicum in Bydgoszcz, Nicolaus Copernicus University in Toruń, K. Ujejskiego St. 75, 85-168 Bydgoszcz, Poland; 5 Institute of Exact and Technical Sciences, Pomeranian University in Słupsk, Arciszewski St. 22B, 76-200 Słupsk, Poland; vladimir.tomin@upsl.edu.pl; 6 Department of Nature Conservation, Institute of Biological Sciences, University of Zielona Góra, Prof. Z. Szafran St. 1, 65-516 Zielona Góra, Poland

**Keywords:** microplastics, nanoplastics, early-life exposure, sorption mechanisms, gut barrier development, developmental toxicology

## Abstract

Microplastics and nanoplastics (MNPLs) are increasingly recognized as dynamic vectors capable of transporting a wide range of environmental contaminants, as well as acting as physical particulates. Their small size, high surface reactivity and strong sorption capacity allow them to carry metals, pesticides, pharmaceuticals and endocrine-active compounds into biological systems. This narrative review examines how these particle-contaminant complexes influence oxidative stress, inflammatory signaling and endocrine function during early development. Relevant literature was identified through structured searches of PubMed, Scopus, Web of Science and Google Scholar, with a focus on the physicochemical properties of plastics, sorption mechanisms, gut barrier physiology and developmental toxicology. Early developmental stages are particularly sensitive, as immature mucus layers, permeable epithelial junctions and underdeveloped detoxification pathways facilitate the uptake and systemic distribution of MNPLs. Once internalized, these particles and their chemical cargo promote the generation of reactive oxygen species through redox-active contaminants, surface-catalysed reactions and mitochondrial dysfunction. The resulting oxidative imbalance activates stress-responsive pathways, including Nrf2–Keap1 signaling, and promotes lipid peroxidation, DNA damage and cellular dysfunction. MNPLs also stimulate inflammatory cascades by activating pattern-recognition receptors, altering cytokine profiles and disrupting epithelial homeostasis. These responses are intensified in the presence of sorbed pollutants, leading to sustained inflammatory states that can be particularly detrimental during organogenesis and immune maturation. Endocrine function is likewise affected, as MNPLs transport hormonally active chemicals and can interfere with hormone-responsive pathways through oxidative and inflammatory mechanisms. These interactions may disrupt thyroid signaling, metabolic regulation and the development of the reproductive axis, with potential long-term physiological consequences. Integrating evidence from polymer chemistry, contaminant behavior and developmental physiology, this review shows that MNPLs act as biologically active vectors that may increase oxidative, inflammatory and endocrine disturbances during early development. These findings highlight the importance of considering particle–contaminant interactions as a critical component of early-life risk assessment.

## 1. Introduction

MNPLs are widespread contaminants in terrestrial, freshwater and marine ecosystems. The presence of MNPLs in food, drinking water and atmospheric fallout is an increasingly recognized public health concern [1]. Due to their small size, persistence and physicochemical properties, MNPLs can readily interact with biological systems and accumulate in various tissues, raising concerns about their potential long-term impact on human health. Developing organisms are particularly vulnerable to environmental toxicants due to their immature detoxification systems, rapidly proliferating tissues, and heightened metabolic demands. This has long been recognized as a major focus of developmental toxicology research [2]. Furthermore, exposure to environmental contaminants in early life can induce persistent molecular and physiological alterations that persist into adulthood, thereby increasing susceptibility to chronic diseases later in life.

In recent years, MNPLs have been recognized as not only physical stressors, but also active vectors capable of adsorption, concentrating, and transporting environmental pollutants. This role has been increasingly emphasized in studies demonstrating their ability to carry perfluoroalkyl substances (PFAS), metals, and other contaminants across biological and environmental interfaces [3,4,5,6]. Kovacs et al. [7] have emphasized that these particles readily bind to endocrine-disrupting chemicals, heavy metals, and persistent organic pollutants, thereby substantially modifying their toxicokinetics. The large surface area to volume ratio and hydrophobic nature of many MNPLs facilitate the adsorption of lipophilic contaminants, thereby enhancing their environmental mobility and potential bioavailability.

This vector-like behavior can significantly alter the internal dose and tissue distribution of co-occurring chemicals, including pesticides, pharmaceuticals, and PFAS, many of which are recognized developmental toxicants [8]. Evidence from toxicological studies suggests that contaminants absorbed into the gastrointestinal tract can subsequently desorb and cross the immature intestinal barrier. There, they interact with molecular pathways that regulate oxidative balance, inflammatory signaling and endocrine homeostasis [9]. Furthermore, MNPLs themselves may induce epithelial barrier dysfunction, mitochondrial impairment, and gut microbiota dysbiosis, thereby exacerbating the toxicity of associated contaminants. The disruption of pathways such as the Nrf2-Keap1 antioxidant signaling pathway, the NF-κB inflammatory cascade and the MAPK signaling pathway, as well as hormone receptor-mediated endocrine regulation, during early development can lead to long-term physiological and metabolic consequences [10]. Therefore, MNPLs should not be regarded merely as inert environmental particles, but as biologically active carriers that may increase the developmental toxicity of co-existing environmental pollutants.

The biological relevance of this phenomenon is substantial. MNPLs have been shown to cross important biological barriers, including the intestinal epithelium, the placenta, and the blood–brain barrier. This raises serious concerns about their systemic distribution during early development [11]. This ability is particularly alarming during the prenatal and early postnatal periods, when organ systems are still developing and are therefore highly susceptible to environmental insults. Pannetier et al. [12] also demonstrated that pollutants adsorbed onto MNPLs in the environment can exert cytotoxic and oxidative effects once internalized. Basu et al. [13] have argued that such interactions may increase molecular stress responses during sensitive developmental periods, thereby raising the risk of chronic diseases in adulthood. Furthermore, emerging evidence suggests that chronic exposure to MNPLs may contribute to immune dysregulation, metabolic disturbances, and neurodevelopmental alterations by persistently activating inflammatory and oxidative pathways.

Although oral ingestion is considered the primary route of human exposure, current estimates of annual intake (39,000–52,000 particles) are based on indirect assessments and limited by analytical sensitivity. Commonly consumed products such as table salt, seafood, and bottled water have been shown to contain microplastics. However, most quantitative data derive from environmental monitoring rather than direct human biomonitoring [14]. Inhalation and dermal exposure have been proposed as additional routes of exposure, yet dietary intake remains the dominant pathway in most populations. There is well-established evidence for trophic transfer in animal models, including the study by Li et al. [15], which demonstrated the unintentional ingestion of microplastics by fish and their subsequent movement through aquatic food webs. However, such findings suggest the potential for human uptake rather than confirming it.

Regarding internal distribution, only particles very small—typically in the sub-5 µm or nanometre range—have been shown in in vitro and animal studies to cross the intestinal epithelium and enter circulation. Reports of microplastics in human tissues (e.g., the liver, spleen, kidneys and placenta) [16] are based on small sample sizes and rely on spectroscopic detection at the limit of current resolution. They do not yet allow firm conclusions to be drawn about dose, polymer type or exposure route. Therefore, these findings represent emerging evidence rather than definitive proof of systemic migration in humans. Due to their smaller size and larger reactive surface area, MNPLs may show increased tissue penetration and greater biological reactivity compared with larger plastic particles. Despite the rapidly expanding body of research, the combined effects of MNPLs and their associated contaminants remain insufficiently characterized, particularly in the context of developing organisms and long-term developmental toxicity.

As demonstrated by Santhanam et al. [3], oxidative stress initiated by metals and influenced by pesticide metabolism may increase the reactive oxygen species (ROS)-generating effects of perfluoroalkyl substances (PFAS) transported by microplastics. In addition to their direct oxidative effects, microplastics act as efficient carriers, concentrating and delivering PFAS and other persistent pollutants into biological systems, thereby contributing to increased toxic outcomes across species and tissues [4]. This type of co-exposure can overwhelm the body’s defense mechanisms, leading to lipid peroxidation, protein oxidation, mitochondrial dysfunction and DNA damage. Studies using aquatic models demonstrate that PFAS-coated microplastics can disrupt photosynthesis, antioxidant defenses and membrane integrity. For example, similar results were observed in *Chlorella sorokiniana* exposed to PFOA and MNPLs [17], as well as in submerged aquatic plants exposed to PFAS alongside UV-aged PLA particles [18]. These findings suggest that the interaction between microplastics and co-existing pollutants can produce complex mixture effects that may exceed the toxicity of individual contaminants.

These interactions are not merely ecological observations. As highlighted in reviews by Parashar et al. [19] and Santhanam et al. [3], PFAS readily adsorb onto microplastic surfaces, increasing their bioavailability and facilitating cellular uptake. The efficiency of adsorption depends on particle size, polymer composition, surface charge, ageing processes and environmental conditions such as pH and salinity. All of these factors influence the binding and release of contaminants. At the cellular level, PFAS induce ROS generation through both mitochondrial and non-mitochondrial pathways, reducing catalase activity and triggering lysosomal leakage and lipid peroxidation, as demonstrated by Amstutz et al. in HepG2 cells [20]. These processes contribute to mitochondrial dysfunction, impaired energy metabolism and the activation of apoptosis-related pathways, thereby increasing cellular injury under co-exposure conditions.

Notably, studies investigating mixture toxicity report that PFAS and MNPLs may act in a way that increases ROS production, DNA damage and redox-related gene activation across multiple human cell lines [21], and may intensify oxidative stress responses in sentinel species such as *Daphnia magna* [22] and zebrafish embryos [23]. These findings indicate that microplastics can influence not only contaminant transport, but also the magnitude and persistence of toxicological responses at the molecular and cellular levels.

These observations highlight that microplastics carry PFAS and reshape exposure dynamics. They also enhance cellular penetration and create biochemical conditions that may increase PFAS-driven oxidative stress, genotoxicity and developmental toxicity. As stated in broader PFAS toxicology frameworks [24,25], such mixture effects complicate the interpretation of traditional single-compound risk assessments, which often do not account for interactions between particulate carriers and sorbed contaminants. Emerging evidence also suggests that simultaneous exposure to microplastics and PFAS may influence gene expression patterns and epigenetic regulation during critical developmental periods, potentially contributing to long-term susceptibility and transgenerational effects [26]. This narrative review aims to provide an integrated overview of how micro- and nanoplastic particles act as carriers of environmental contaminants, and of the influence of this combined exposure on molecular pathways during early development. Particular emphasis is placed on the mechanisms of contaminant sorption and intestinal transport, and the subsequent activation of oxidative, inflammatory, and endocrine signaling networks. The central role of oxidative stress in connecting contaminant transport with downstream cellular dysfunction and developmental impairment is also examined in detail. The review’s specific contribution lies in connecting physicochemical interactions occurring at the MNPL surface with defined molecular pathways disrupted in developing organisms, thereby distinguishing vector-mediated exposure from exposure to contaminants alone. By integrating concepts from environmental chemistry, developmental physiology and molecular toxicology, the review outlines emerging paradigms in mixture toxicology and identifies key knowledge gaps that require further investigation.

## 2. Literature Search and Study Selection

A comprehensive literature search was conducted in PubMed, Scopus, Web of Science, and Google Scholar to identify studies examining the interactions between MNPLs and co-occurring environmental contaminants. Searches were performed between January and March 2025 and covered publications from 2014 to 2025. In PubMed, the following Title/Abstract search string was used: (“microplastics” [Title/Abstract] OR “nanoplastics” [Title/Abstract]) AND (“oxidative stress” [Title/Abstract] OR “inflammation” [Title/Abstract] OR “epithelial barrier” [Title/Abstract] OR “endocrine disruption” [Title/Abstract] OR “development” [Title/Abstract]) AND (“contaminant” [Title/Abstract] OR “metal” [Title/Abstract] OR “pesticide” [Title/Abstract] OR “endocrine-active” [Title/Abstract] OR “xenobiotic” [Title/Abstract] OR “sorption” [Title/Abstract] OR “desorption” [Title/Abstract]), with filters restricted to English-language, peer-reviewed publications published between 2014 and 2025. In Scopus, the search syntax was: (TITLE-ABS-KEY (“microplastics” OR “nanoplastics”) AND TITLE-ABS-KEY (“oxidative stress” OR “inflammation” OR “epithelial barrier” OR “endocrine disruption” OR “development”) AND TITLE-ABS-KEY (“contaminant” OR “metal” OR “pesticide” OR “endocrine-active” OR “xenobiotic” OR “sorption” OR “desorption”)) AND (PUBYEAR > 2013 AND PUBYEAR < 2026) AND (LIMIT-TO (LANGUAGE, “English”)). In Web of Science, the Topic Search query was: TS = ((“microplastics” OR “nanoplastics”) AND (“oxidative stress” OR “inflammation” OR “epithelial barrier” OR “endocrine disruption” OR “development”) AND (“contaminant” OR “metal” OR “pesticide” OR “endocrine-active” OR “xenobiotic” OR “sorption” OR “desorption”)), refined by publication years (2014–2025), document type (Article), and language (English). Due to platform-specific limitations, Google Scholar searches were conducted using simplified queries (“microplastics” “oxidative stress” “development”; “nanoplastics” “endocrine disruption”; and “microplastics” “inflammation” “epithelial barrier”), and only the first 200 results ranked by relevance were screened.

Studies were considered eligible if they investigated interactions between MPs or NPs and co-occurring contaminants, including metals, pesticides, per- and polyfluoroalkyl substances (PFAS), endocrine-active compounds, or other xenobiotics, and reported outcomes related to early-life exposure, toxicokinetics, cellular signaling, oxidative stress, immune dysregulation, endocrine disruption, or developmental effects. Both in vivo and in vitro studies were included, encompassing vertebrate and invertebrate models to capture conserved physiological and molecular responses across taxa. Studies focusing exclusively on isolated contaminants without concurrent MP or NP exposure, publications lacking biological endpoints, conference abstracts, reviews, editorials, and non-peer-reviewed sources were excluded from the analysis.

The search yielded 1243 records across all databases. After the removal of 217 duplicate records, 1026 publications underwent title and abstract screening. Subsequently, 412 full-text articles were assessed for eligibility, and 292 studies met the predefined inclusion criteria and were incorporated into the qualitative synthesis. No formal quality appraisal tool was applied. The final body of evidence was systematically analyzed to identify recurring mechanistic pathways linking MP- and NP-mediated contaminant transport with oxidative stress, inflammation, epithelial barrier dysfunction, endocrine alterations, and developmental vulnerability.

The final body of literature was analyzed to identify recurring pathways linking MNPLs -associated contaminant transport with oxidative stress, inflammation, epithelial barrier dysfunction, endocrine alterations, and developmental vulnerability. Studies focusing solely on isolated contaminants without MNPLs co-exposure, papers lacking biological endpoints, and non-peer-reviewed sources were excluded. Both vertebrate and invertebrate models were considered in order to capture conserved physiological and molecular responses across taxa.

## 3. Physicochemical Features and Sorption Capacity of MNPLs

The physicochemical properties of MNPLs are key factors in determining how these particles interact with developing organisms and influence toxicological outcomes. Kovacs et al. [7] emphasise that particle size is one of the most decisive parameters since nanoscale plastics have an exceptionally high surface-to-volume ratio, increased chemical reactivity, and a greater ability to penetrate biological tissues. This is consistent with in vivo findings reported by Zhang et al. [9], who demonstrated that NPs cross epithelial and endothelial barriers far more efficiently than larger MPs. Their small size also enables them to be taken up by cells through endocytosis and passive diffusion mechanisms, thereby increasing the likelihood of subcellular interactions with mitochondria, lysosomes and nuclei. Notably, Deng et al. [27] provided early experimental confirmation that MPs can accumulate in mammalian tissues, indicating that even short-term exposure may result in systemic distribution and organ deposition. The persistence of these particles within tissues raises further concerns regarding chronic inflammation, oxidative imbalance and long-term bioaccumulation.

Developing organisms are uniquely vulnerable to these small particles. As Han et al. [28] reported, immature epithelial barriers are more permeable, enabling particles measuring less than 5 µm to more easily cross the intestinal wall than in adults. Similarly, Feng et al. [29] demonstrated that mucus layers and microbiomes in early life are structurally and functionally immature, further facilitating particle adhesion, retention, and uptake. Reduced mucus production, incomplete tight-junction development, and altered microbial diversity may weaken intestinal defense mechanisms during critical developmental periods. Studies investigating the distribution of microplastics in human tissue provide emerging but still limited evidence of internal accumulation. For example, reports such as that by Zhu et al. [30] describe the presence of microplastics in multiple human organs. However, these findings are based on small cohorts and rely on spectroscopic methods operating near their detection limits. This restricts the ability to draw conclusions regarding particle size, polymer identity, and exposure route. Li et al. [31] proposed potential migration through lymphatic and circulatory pathways, but this is largely inferred from animal and in vitro models rather than demonstrated directly in humans.

Also, evidence for placental transfer is mixed. While MNPLs have been shown to cross the placental barrier and alter nutrient transport, immune signaling and redox balance in in vitro trophoblast models and animal studies, human data remains preliminary and is limited by analytical sensitivity. Taken together, these observations indicate that particle size, particularly within the sub-5 µm and nanoscale range, is a critical determinant of uptake, biodistribution, and developmental susceptibility; however, the strength of the evidence varies substantially across study types.

Surface charge is another critical factor that shapes MNPLs-cell interactions. According to Ullah et al. [10], positively charged MNPLs strongly interact with negatively charged cell membranes, tight-junction proteins and mucosal components. This increases the likelihood of epithelial disruption and enhanced cellular uptake. These electrostatic interactions can destabilise membrane integrity, alter ion transport, and enable the movement of associated contaminants across biological barriers. Tyc et al. [32] also demonstrated that MNPLs carrying endocrine-disrupting additives, such as phthalates, can interact with hormone receptors and exacerbate endocrine disruption. Conversely, Sellami et al. [33] showed that negatively charged or neutral particles preferentially bind metal ions and persistent organic pollutants, thereby modifying their environmental persistence and toxicokinetic behavior. Surface ageing processes, including oxidation and UV-induced weathering, may also alter particle charge and surface chemistry, thereby influencing contaminant sorption and biological interactions.

Hydrophobicity introduces an additional layer of complexity to MNPLs-mediated toxicity. Polymers such as polystyrene and polyethylene readily absorb lipophilic contaminants, as Pannetier et al. [12] documented in environmental MNPLs collected from coastal environments. Basu et al. [13] argued that this high sorption capacity effectively transforms MNPLs into “toxic cocktails”, combining intrinsic polymer additives with environmentally acquired pollutants. This substantially increases the probability of simultaneous exposure to multiple toxicants, complicating toxicological responses and mixture interactions. These observations align with the broader toxicological interactions outlined by Bhagat et al. [8], who emphasized that hydrophobic MNPLs often act synergistically with co-occurring contaminants, leading to increased cellular stress responses, oxidative damage, and inflammatory signaling. Furthermore, hydrophobic interactions facilitate the prolonged retention of contaminants on particle surfaces, potentially enabling sustained release after internalization into biological systems. Therefore, surface charge and hydrophobicity jointly determine how MNPLs adhere to biological surfaces, the contaminants they transport, and their interaction with developing tissues. These physicochemical properties are thus central considerations in environmental and developmental risk assessment [8].

Studies emphasise that different polymers exhibit distinct degradation profiles, sorption capacities, and toxicological signatures. For example, the comparative analysis conducted by Zhang et al. [9] evaluated polystyrene, polyethylene, polypropylene, polyvinyl chloride (PVC), and polyethylene terephthalate (PET), and demonstrated that each polymer type fragments differently and contains unique chemical additives that may influence biological activity and environmental persistence. The analysis revealed that each polymer type fragments differently and contains unique chemical additives that can affect biological activity and environmental persistence. Differences in crystallinity, polymer density and surface morphology also affect the adsorption of contaminants and the environmental fate of individual plastic types. These additives, including plasticisers, stabilisers, flame retardants and coloring agents, may subsequently leach into biological systems under physiological or environmental conditions. In their 2025 study, Tyc et al. [32] documented endocrine disruption across multiple hormonal axes, including the hypothalamic-pituitary-gonadal (HPG), hypothalamic-pituitary-thyroid (HPT) and hypothalamic-pituitary-adrenal (HPA) systems. Meanwhile, Sellami et al. [33] demonstrated that certain additives associated with plastics bind oestrogen receptors with high affinity, thereby interfering with endocrine signaling pathways. Such interactions may alter hormonal homeostasis during sensitive developmental periods, potentially contributing to reproductive, metabolic and neurodevelopmental disturbances later in life.

Another important factor is that polymer surfaces are continuously modified by environmental weathering, which consequently alters their biological behavior. Oxidation, UV exposure and mechanical abrasion introduce oxygen-containing reactive functional groups, increase surface roughness and promote fragmentation into nanoscale particles [34]. These weathering processes also make polymers more brittle and increase the release of secondary particles, which are more mobile and biologically reactive than larger particles. Bhagat et al. [8] demonstrated that weathered MPs exhibit a markedly higher sorption capacity for environmental pollutants, while Kovacs et al. [7] observed that weathering increases the potential for oxidative stress and inflammatory responses in biological tissues. Additionally, aged particles may exhibit altered surface charge and hydrophilicity, thereby altering their interactions with proteins, membranes, and cellular receptors. Therefore, the evolving physicochemical identity of MPs and their toxicological properties are influenced by polymer chemistry and environmental ageing long before these particles enter the human body.

Once inside a biological environment, MNPLs rapidly acquire a biocorona, which is composed of proteins, lipids, polysaccharides, and microbial components derived from the surrounding biological fluids. Kovacs et al. [7] described this process as a fundamental transformation that alters the particle’s charge, hydrophobicity, and cellular recognition. Biocorona formation creates a new biological interface that determines how cells and tissues perceive and respond to the particle. Furthermore, Feng et al. [29] noted that biocorona composition differs markedly between infants and adults due to age-specific serum proteins, immune mediators, and microbiome profiles. Consequently, the same particle may behave differently depending on developmental stage and physiological environment. Zhu et al. [30] and Li et al. [31] both demonstrated that biocorona formation significantly influences tissue distribution, with those formed during early life promoting deeper tissue penetration and prolonged retention. Emerging evidence also suggests that biocoronas may modulate immune activation, inflammatory signalling, and the intracellular trafficking of associated contaminants. These findings emphasise that MNPLs are dynamic particles whose biological identity evolves continuously, thereby complicating toxicological predictions and risk assessment.

Exposure pathways determine which physicochemical properties are most relevant to internalization and toxicity. Oral ingestion remains the dominant route of exposure. Cox et al. [1] estimated that adults ingest approximately 39,000–52,000 MNPLs annually. However, Zuri et al. [35] reported even higher exposure levels when inhalation was included in exposure calculations. Significant dietary sources include oysters [36], mussels [37,38], fish [15,16], table salt [14,39], honey [40] and bottled water [39]. Food packaging materials and processing technologies are also increasingly recognized as major contributors to dietary MNPL contamination.

Household practices are another important contributor to human exposure. For instance, Hussain et al. [41] showed that microwaving plastic containers releases millions of MNPLs, and Ranjan et al. [42] found that disposable paper cups release tens of thousands of particles into hot liquids. Li et al. [43] also identified mobile phone cases as a previously unrecognized source of chronic MNPLs shedding. Synthetic textiles, indoor furnishings, and household dust are also increasingly recognized as significant sources of airborne MNPLs in indoor environments. Importantly, infants and children appear to be disproportionately exposed. Studies investigating indoor dust have reported extremely high concentrations of MNPLs [44], and Mišľanová et al. [45] have estimated that infants ingest nearly twice as many MNPLs as adults. Breast milk contamination, as documented by Saraluck et al. [46], further highlights the vulnerability of infants and young children during critical developmental periods.

Although current evidence for internal distribution following respiratory uptake remains limited, inhalation is increasingly recognized as a potentially important exposure route. Reports such as Weingrill et al. [11], which detected microplastics in human placental tissues, and Leonard et al. [47], which identified multiple polymer types in human blood, provide emerging human evidence, but these findings rely on small sample sizes and analytical methods operating near their detection limits. Consequently, firm conclusions regarding particle size, polymer identity, and the specific contribution of inhalation relative to other exposure routes cannot yet be drawn.

Insights into the mechanisms of respiratory uptake largely come from in vitro airway models and animal studies. These studies show that airborne fibres and ultrafine plastic particles, particularly those in the sub-5 µm and nanoscale range, can penetrate deep into the respiratory tract. They can also induce oxidative stress, trigger local inflammatory responses, and disrupt pulmonary barrier integrity. Dermal exposure is generally considered to be less efficient than ingestion or inhalation. However, in vitro skin models suggest that nanoscale particles may interact with the epidermal barrier under certain conditions. This indicates that this exposure route requires further investigation.

MNPLs present in cosmetics [48] and skin-cleansing products [49] may accumulate on the skin’s surface. Meanwhile, additives such as phthalates and triclosan can penetrate the epidermis [50]. Repeated low-dose dermal exposure may therefore contribute to the accumulation of plastic-associated chemicals in the body, particularly under conditions of prolonged contact or impaired skin barrier integrity.

Studies by Rosenfeld [51] have demonstrated that the hypothalamus is particularly sensitive to environmental toxicants. Bisphenol A (BPA) and bisphenol S (BPS), for example, have been shown to reduce populations of hypothalamic neurons and alter neuroendocrine signaling. Given the hypothalamus’s central role in regulating energy balance, reproduction, circadian rhythms, and stress responses, disruption to its development could have profound and long-lasting physiological consequences. Furthermore, Sharma et al. [52] reported neurobehavioral alterations following NP exposure, while Gao et al. [53] and Baroni et al. [54] linked MNPLs to cognitive impairment, behavioral abnormalities, and altered neurotransmission. Furthermore, experimental studies suggest that MNPLs may induce neurotoxicity through oxidative stress, mitochondrial dysfunction, neuroinflammation, and disruption of the blood-brain barrier. Endocrine disruption associated with plastic particles is also well documented. Graceli et al. [55], He and Yin [56] demonstrated that MNPLs interfere with the hypothalamic-pituitary-gonadal (HPG), hypothalamic-pituitary-thyroid (HPT) and hypothalamic-pituitary-adrenal (HPA) axes, thereby affecting hormonal homeostasis across multiple physiological systems. Disturbances within these axes during early development may impair reproductive maturation, metabolic regulation and adaptive stress responses later in life. Ullah et al. [10] argued that future research should focus specifically on the direct effects of MNPLs on hypothalamic nuclei due to their pivotal role in coordinating metabolism, endocrine function and stress regulation.

These findings demonstrate that once MNPLs enter biological or environmental systems, they undergo a series of transformations that fundamentally alter their identity and toxicological behavior. The rapid formation of a biocorona, composed of proteins, lipids, polysaccharides and microbial molecules, modifies particle charge, hydrophobicity and cellular recognition, thereby influencing adhesion, uptake and intracellular trafficking, as demonstrated by Kovacs et al. [7]. Biocorona formation may additionally shield or expose reactive particle surfaces, thereby modulating immune recognition and inflammatory activation. Importantly, the composition of the biocorona differs between early developmental stages and adulthood because of variations in mucus composition, serum proteins and microbiome structure during ontogenesis. These developmental differences may either enhance or restrict particle translocation and tissue accumulation, as highlighted by Feng et al. [29]. Most available studies rely on pristine particles and doses that exceed environmentally relevant concentrations; therefore, these findings should be interpreted with caution when considering early-life exposure.

Environmental weathering processes, including oxidation, UV exposure and mechanical abrasion, modify the surfaces of MNPLs by increasing their roughness and generating reactive oxygen-containing functional groups. These alterations promote the fragmentation of MNPLs into highly reactive nanoscale particles with an enhanced capacity to bind environmental contaminants, as observed by Bhagat et al. [8]. Weathered particles also exhibit altered surface chemistry and increased environmental persistence; these factors may intensify their interactions with biological membranes and intracellular components. Such aged particles may provoke stronger oxidative and inflammatory responses in developing tissues, a vulnerability that is further supported by human tissue distribution studies conducted by Zhu et al. [30]. Meanwhile, polymer-embedded additives, including plasticisers, stabilisers, and flame retardants, may leach from the particle matrix. This introduces additional intrinsic endocrine-disrupting and pro-oxidant effects, which intensify the toxicity of sorbed pollutants [32]. The simultaneous presence of sorbed contaminants and leached additives may therefore create complex mixture effects capable of disrupting multiple molecular pathways simultaneously. These interconnected processes generate a continuously evolving MNPLs identity that amplifies biological reactivity, enhances toxicological complexity, and increases susceptibility to adverse outcomes during early development.

### 3.1. Polymer Type and Its Relevance to Early-Life Exposure

It is essential to understand how different polymers behave in biological systems when evaluating the risks associated with MNPLs during early development. This is because early life stages are characterized by immature detoxification systems, elevated metabolic demands and high ingestion rates relative to body mass [57]. Tokiwa et al. [57] demonstrated that the chemistry, crystallinity, density and composition of additives in polymers fundamentally determine their fragmentation, degradation and sorption behavior, which directly influences the exposure profile of embryos and larvae. Differences in polymer structure also influence particle persistence, surface reactivity, and the capacity to absorb environmental contaminants, thereby affecting the biological availability of toxic compounds. Chanda et al. [5] further reinforced this concept, emphasizing that polymer morphology, density, and weathering state govern transport, settling behavior, and bioavailability across aquatic ecosystems. This ultimately influences the types of particles encountered by developing organisms during sensitive developmental periods. Therefore, polymer-specific properties are key determinants of developmental exposure and toxicological susceptibility.

Pielichowski et al. [58] reported that polystyrene (PS) is highly hydrophobic and prone to forming nanoscale fragments with smooth yet reactive surfaces. The aromatic ring structures of PS also facilitate strong π–π interactions with hydrophobic contaminants. These properties make PS an efficient carrier for pesticides, pharmaceuticals, and endocrine-active compounds, thereby increasing the chemical burden delivered to developing tissues [58]. The enhanced sorption of lipophilic toxicants onto PS surfaces may prolong contaminant retention and increase the likelihood of sustained exposure after internalization. Furthermore, Lerman et al. [59] demonstrated that PS requires surface oxidation to support cell adhesion in vitro, which indirectly highlights its chemically inert yet highly interactive surface and strong sorptive potential. Furthermore, Pfaff et al. [60] showed that PS nanospheres can be functionalized with glycopolymers, demonstrating their capacity to bind readily to biological macromolecules. In environmental settings, this property enables the swift formation of biocoronas, which can alter cellular uptake and immune recognition. Rübsam et al. [61] also demonstrated that peptides can evolve to bind to PS surfaces with high affinity. This further confirms that PS particles are biologically interactive and chemically accessible. These findings suggest that PS particles may not only serve as passive carriers of contaminants, but also as dynamic interfaces capable of modulating cellular signaling and molecular interactions during development.

Studies by Majewsky et al. [62] have shown that polyethylene (PE) and polypropylene (PP) remain suspended in the water column due to their low density. This increases the likelihood of their being ingested by planktonic organisms during the early stages of development. This is important because prolonged suspension increases the probability of repeated encounters during critical developmental periods [62]. Their widespread environmental persistence also leads to continuous low-dose exposure across aquatic food webs. Glaser et al. [63] demonstrated that environmental oxidation introduces carbonyl and hydroxyl groups onto the surfaces of PE and PP, thereby increasing surface polarity and promoting biocorona formation. Such modifications may alter immune recognition, gastrointestinal retention time, and interactions with epithelial barriers.

A 2023 study by Wróbel et al. [64] revealed that PP undergoes more extensive microcracking during microbial degradation than PE, suggesting greater susceptibility to fragmentation into smaller, more reactive particles. In a separate investigation, Lv et al. [65] confirmed that microbial colonization accelerates oxidative erosion, leading to an increased surface area and enhanced physicochemical reactivity. These ageing processes can substantially increase the adsorption of metals, PFAS (per- and polyfluoroalkyl substances) and persistent organic pollutants onto PE and PP surfaces, thereby intensifying their vector potential. Jolaosho et al. [6] further emphasized that PE and PP dominate MNPLs profiles in both freshwater and marine environments globally, highlighting the importance of polymer-specific behaviors in the context of developmental exposure and early-life toxicology. Due to their abundance and persistence, particles derived from PE and PP are likely to be one of the most significant sources of chronic MNPLs exposure during prenatal and postnatal development.

Analysis by Yu et al. [66] revealed that polyvinyl chloride (PVC) contains high levels of plasticisers, particularly phthalates. These plasticisers readily leach from the polymer matrix and exhibit potent endocrine-disrupting properties. This is particularly concerning during the early stages of development, when tightly regulated endocrine signaling controls organogenesis, growth, and neurodevelopment [66]. Leached additives from PVC may interfere with steroid hormone receptors, thyroid signaling and reproductive development, thereby increasing vulnerability during prenatal and postnatal life. Tuncelli et al. [67] demonstrated that PVC strongly adsorbs cadmium (Cd) and that exposure to a combination of PVC MNPLs and Cd results in increased oxidative stress, apoptosis and metabolic disruption in mussels. This highlights the importance of understanding interaction effects in mixture toxicity. Li et al. [68] corroborated these findings through a meta-analysis demonstrating that MNPLs act as oxidative stressors in bivalves, with combined exposure resulting in greater physiological disturbance than exposure to a single toxicant. Furthermore, Ren et al. [69] and Sha [70] reported that PVC is among the polymers most frequently associated with developmental malformations, oxidative stress, and behavioral abnormalities in zebrafish, which are an important vertebrate model for early-life toxicology. These findings suggest that PVC particles may be a particularly hazardous type of MNPLs because they combine intrinsic chemical toxicity with the ability to transport co-occurring pollutants.

Thomaz et al. [71] explained that, despite being more polar than polystyrene (PS) or polyethylene (PE), polyethylene terephthalate (PET) can efficiently absorb perfluoroalkyl substances (PFAS), pharmaceuticals, and metal ions via hydrogen bonding and electrostatic interactions. This is highly relevant because PET is widely used in containers for bottled water and infant-feeding products, making it a common and realistic source of early-life exposure [70]. Repeated exposure through feeding bottles, food packaging, and heated beverages may therefore substantially contribute to chronic, low-dose PET exposure during infancy. Kauts et al. [72] demonstrated that PET MNPLs accumulate in the digestive tract of *Drosophila melanogaster*, impairing locomotion and neuromuscular function. Meanwhile, Liang et al. [73] showed that long-term exposure to PET particles produces sex-specific effects on lifespan, including hormetic responses at low doses. Brito et al. [74] also reported that PET degradation is strongly influenced by autocatalytic hydrolysis and local microenvironmental pH conditions. These processes may alter the surface chemistry of particles and promote the release of monomers and degradation by-products that may have endocrine and metabolic activity. Consequently, the physicochemical transformation of PET within biological and environmental systems may significantly impact its toxicological behavior in developing organisms.

**Figure 1:**
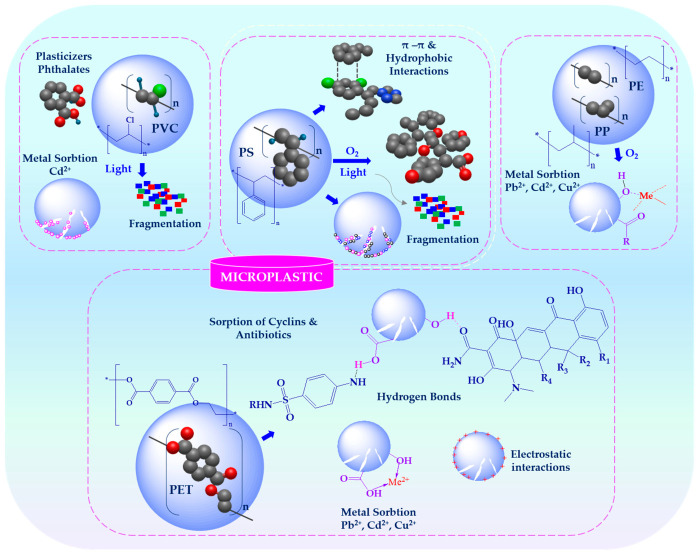
The influence of polymer type and environmental processes on the properties of MNPLs. Polymer type and environmental weathering determine the reactivity, contaminant interactions, and toxicity of MNPLs. PS strongly sorbs organic pollutants, PE and PP become more reactive after oxidation, PVC releases and transports toxic additives, and PET adsorbs contaminants and may release monomers during degradation. Weathering processes enhance bioavailability, biocorona formation, contaminant transport, and toxicological effects, especially during early development. Abbreviations: PVC–poly(vinyl chloride); PE–polyethylene; PET–poly(ethylene terephthalate); PP–polypropylene; PS–polystyrene.

Figure 1 illustrates how different types of MNPLs exhibit distinct physicochemical properties that determine their interaction with environmental contaminants and biological systems. It also shows how weathering processes in the environment modify MNPLs surfaces, making them more reactive and increasing their capacity to bind contaminants, form biocoronas and have toxic effects, particularly during early developmental stages.

Further studies have demonstrated that degradation behavior substantially modulates polymer-specific risks. Pielichowski et al. [58] clarified that PS and PVC fragment into smaller, more reactive particles under UV radiation, thereby increasing their bioavailability and capacity to cross epithelial barriers. The formation of nanoscale fragments is important because smaller particles can penetrate tissue more easily, are taken up more readily by cells and are more reactive. By contrast, PE and PP undergo surface oxidation rather than extensive fragmentation, generating functional groups that enhance contaminant sorption and facilitate biocorona formation [63]. Jiao et al. [75] also showed that although PET is relatively resistant to fragmentation, it can release monomers and oligomers that can interfere with metabolic and endocrine pathways. Both Chanda et al. [5] and Jolaosho et al. [6] emphasized that environmental weathering, heteroaggregation, and additive leaching can fundamentally alter polymer behavior, influencing the exposure routes encountered by early-life organisms. Consequently, weathered particles may exhibit toxicological properties that differ substantially from pristine plastics, complicating the assessment of hazards and the prediction of biological effects.

These studies demonstrate that the type of polymer determines how MNPLs behave physically and chemically within environmental and biological systems. They also show that MNPLs can act as vectors for other pollutants and modify oxidative and inflammatory signaling pathways, disrupting endocrine regulation during early development [57,68]. The interaction between intrinsic polymer properties, associated additives, and sorbed environmental contaminants creates complex mixture effects that can influence multiple physiological systems simultaneously. Understanding these polymer-specific mechanisms is essential for the accurate assessment of ecological and developmental risks, as well as for developing effective mitigation strategies aimed at protecting vulnerable early life stages [70,76,77].

Table 1 provides a comparative summary of polymer-specific physicochemical characteristics, degradation pathways, and early-life toxicological outcomes. The table integrates evidence from aquatic and terrestrial vertebrate and invertebrate model systems to highlight conserved mechanisms of polymer-specific developmental toxicity. Physicochemical ageing processes, including oxidation, fragmentation and biocorona formation, are included where relevant to exposure dynamics during early developmental stages.

Furthermore, the intrinsic properties of each polymer type, such as differences in hydrophobicity, crystallinity, surface chemistry and additive content, have a significant impact on early-life exposure to MNPLs. These physicochemical characteristics influence the environmental fate of particles (e.g., transport, sedimentation and persistence) and their ability to undergo ageing processes, such as oxidation, biofilm formation and fragmentation into micro- and nanoscale particles [82]. These properties influence the interaction of particles with environmental contaminants, their weathering and fragmentation, and their uptake by developing organisms. In particular, surface reactivity and the balance between hydrophobic and hydrophilic properties govern the efficiency of contaminant sorption, while particle size and morphology are key determinants of epithelial penetration and systemic translocation [6].

Crucially, these polymer-specific traits result in different toxicological profiles in various biological models, such as mussels, zebrafish and *Drosophila* [83,84,85]. Evidence suggests that MNPLs can cause oxidative stress, metabolic disruption, developmental abnormalities, and immune or reproductive impairment. These effects are mediated through common pathways, such as ROS overproduction, mitochondrial dysfunction, inflammatory signaling (e.g., NF-κB activation) and disruption of the endocrine axis [7]. However, the relative contribution of each pathway varies depending on the type of polymer, the organism’s age and any associated contaminants. Therefore, the severity and basis of toxicity vary markedly between polymers, reflecting differences in both intrinsic material properties and environmentally acquired modifications. These findings support the idea that MNPLs should not be considered a uniform contaminant class, but rather a heterogeneous group of materials with polymer-dependent toxicological signatures that are modified by environmental and biological interactions.

### 3.2. The Mechanisms of Sorption of Heavy Metals, Pesticides, Pharmaceuticals and PFAS

The growing prevalence of MNPLs in aquatic and terrestrial environments means they are becoming dynamic vectors that mobilise and redistribute heavy metals, pesticides, pharmaceuticals, and PFAS. Understanding their sorption mechanisms is therefore essential for assessing the risks associated with exposure in early life, when even low-level chronic exposure can have disproportionate developmental effects. Sorbed contaminants may interact with the polymer matrix, thereby modifying the bioavailability, toxicity, and transport across biological barriers. As demonstrated by Liu et al. [86], Pb^2+^ sorption is significantly higher in naturally aged MNPLs due to the formation of oxygen-containing functional groups. This confirms that electrostatic interactions, surface complexation and ionic exchange dominate metal binding in environmentally weathered polymers [86]. This is important because ageing transforms relatively inert plastics into highly reactive carriers that can accumulate metals such as Pb^2+^, Cd^2+^ and Cu^2+^ by coordinating with carbonyl, hydroxyl and carboxyl groups [87]. This significantly increases the environmental persistence and biological availability of metal-plastic complexes.

The sorption of heavy metals onto MNPLs is primarily governed by electrostatic interactions, surface complexation involving functional groups formed during environmental weathering and coordination chemistry processes. Oxidized surfaces enriched in carbonyl, hydroxyl and carboxyl groups have a high affinity for cationic metals, such as Pb^2+^, Cd^2+^ and Cu^2+^, and form stable inner-sphere complexes with them. Wang et al. [88] confirmed that UV-aged PP, PE and PS exhibit 1.25–1.63 times higher sorption of Pb^2+^ and Cu^2+^, demonstrating that oxidation and increased surface hydrophilicity enhance metal binding capacity. These processes are further influenced by environmental parameters such as salinity, ionic strength and pH, which affect the competition between metal ions and other dissolved species [88]. These findings are significant because environmental ageing increases the residence time of metals on microplastic surfaces, facilitating their co-transport into organisms and enabling desorption under acidic gastrointestinal conditions [89].

PVC has a particularly strong capacity to bind metals due to its polar, chlorine-containing backbone. Meanwhile, aged PE and PP acquire negatively charged functional sites that further enhance metal sorption. These interactions increase the residence time of metals on microplastic surfaces and facilitate their co-transport into biological systems, where they may be desorbed under acidic gastrointestinal conditions [90]. Lin et al. [91] also showed that the sorption capacities of PVC, PE and PS differ markedly, with PVC exhibiting the highest Pb^2+^ uptake, followed by PE and PS. This highlights the critical role of polymer polarity, surface chemistry, and backbone composition in governing metal-binding behavior [91]. Furthermore, additives present in PVC (e.g., stabilisers and plasticisers) can enhance complexation processes and modify the surface reactivity of aged particles [92].

Guan et al. [93] demonstrated that aged polyethylene (PE) in sediments exhibits increased Pb^2+^ sorption, whereas aged poly(lactic acid) (PLA) shows reduced sorption capacity. This illustrates that biodegradable and conventional plastics respond differently to environmental ageing processes [93]. Building on this, Chen et al. [94] showed that, in some cases, biodegradable PLA MNPLs can absorb more metals than conventional plastics due to the higher density of oxygen-containing functional groups formed during hydrolytic degradation. This is significant because biodegradable plastics are often considered to be safer for the environment. However, these findings suggest that they may facilitate the accumulation and subsequent release of metals more readily than traditional polymers [95]. These findings challenge the assumption that biodegradability necessarily equates to reduced ecotoxicological risk.

A meta-analysis by Bi et al. [96] confirmed that surface functional groups, pH, and polymer type are the strongest predictors of metal sorption behavior. Pb^2+^ was found to show the highest affinity across all evaluated polymers. Furthermore, He et al. [97] demonstrated that aged PE increases cadmium sorption in rhizosphere biofilms, suggesting that MNPLs can intensify the accumulation and cycling of heavy metals in soil ecosystems. This suggests that MNPLs may act as secondary reservoirs of metals in terrestrial environments, affecting plant uptake and potentially altering the transfer of metals through the food chain [97]. These studies demonstrate that the sorption of heavy metals onto MNPLs is governed by a combination of electrostatic interactions, surface complexation, van der Waals forces, and intra-particle diffusion processes. They also consistently show that environmental ageing amplifies these mechanisms by increasing surface oxidation, roughness, and functional group density, thereby enhancing the overall metal-binding capacity [98]. Consequently, MNPLs should be regarded as dynamic, evolving geochemical interfaces, rather than inert particles. Their sorption behavior is strongly dependent on both polymer type and environmental history [99].

Pesticides and pharmaceuticals interact with MNPLs via a mixture of hydrophobic partitioning, π–π stacking, hydrogen bonding, and van der Waals forces [78,79]. Hydrophobic polymers such as PS and PE exhibit a strong affinity for non-polar pesticides, including organochlorines and pyrethroids, which readily partition into the polymer matrix. Pharmaceuticals with aromatic or heterocyclic structures, such as antibiotics and antidepressants, often engage in π–π interactions with the aromatic rings of PS, resulting in a high sorption capacity. Hydrogen bonding between the functional groups of weathered plastics and the polar groups of pharmaceuticals further stabilises these interactions. These microplastic-pollutant complexes can significantly alter the bioavailability, environmental persistence and toxicokinetics of the compounds involved, particularly in developing organisms with immature detoxification and excretory systems [78,79]. During the early stages of life, this can result in prolonged internal exposure due to slower metabolic clearance and underdeveloped barrier function.

According to Stapleton et al. [100], the sorption of pharmaceuticals, particularly antibiotics, is governed by hydrophobic partitioning, π–π interactions, hydrogen bonding, and van der Waals forces. Environmental weathering can also increase the sorption capacity by up to 171%. This is particularly relevant because pharmaceuticals with aromatic or heterocyclic structures readily interact with polystyrene and other aromatic polymers, thereby increasing their environmental persistence and altering their bioavailability [100]. Newer studies reinforce these findings, demonstrating that aged PET MNPLs exhibit markedly higher sorption of structurally diverse pharmaceuticals, including β-blockers, antidepressants, and sulfonamides. This is due to increased surface oxidation and roughness. Sorption enhancements can reach a factor of two to four, depending on environmental conditions, as demonstrated by Kalaronis et al. [101]. These effects are further modulated by environmental parameters such as temperature, ionic strength, and dissolved organic matter, which influence adsorption and desorption processes.

Similarly, oxidative ageing via UV/H_2_O_2_ treatment significantly increases the sorption of tetracycline and Cd(II) onto PS and PET. H_2_O_2_-aged MNPLs exhibit the greatest affinity due to the presence of abundant carbonyl and hydroxyl functional groups, as demonstrated by Wang et al. [102]. The authors also emphasise that solution chemistry has a strong influence on sorption behavior: higher salinity reduces antibiotic uptake, while pH determines the strength and direction of electrostatic interactions. These mechanisms are particularly important during the early stages of development, when immature detoxification pathways increase susceptibility to microplastic-pharmaceutical complexes. Importantly, combined antibiotic-metal exposure produces non-additive effects. Cd(II) slightly enhances tetracycline sorption at pH 8.0, particularly on aged MNPLs. This indicates that co-contaminants can modulate sorption behavior in ways that cannot be predicted from single-compound systems [102]. Such mixture effects highlight the limitations of single-pollutant risk assessments in environmental toxicology. This is consistent with broader evidence indicating that MNPLs increase the mobility and bioavailability of heavy metals in aquatic and terrestrial systems, thereby intensifying co-toxicity and oxidative stress responses [103].

Although the primary focus of the included studies was not PFAS-specific sorption, Stapleton et al. [100] emphasise that MNPLs can strongly bind to PFAS due to their amphiphilic structure. This involves hydrophobic interactions with fluorinated carbon chains, as well as electrostatic and hydrogen-bonding interactions with polar head groups. Weathering increases PFAS sorption by introducing oxygen-containing functional groups, thereby enhancing binding strength and environmental persistence [100]. Consequently, aged MNPLs can act as long-term reservoirs of PFAS, facilitating their transport across environmental compartments. These insights are consistent with the finding that aged polyethylene MNPLs alter the environmental behavior of agrochemicals. For example, aged polyethylene MNPLs were found to reduce the concentrations of freely dissolved tetracycline and surfactant cations by ~20%, while simultaneously intensifying plant stress responses, including reductions in chlorophylls of up to 30%, decreases in carotenoids of over 20%, and reductions in APx activity of up to 70%. This demonstrates that sorption-driven changes in bioavailability can directly result in biological toxicity [104].

This is particularly important for early development, as PFAS can desorb in the gastrointestinal tract, where bile salts and proteins compete for binding sites, thereby increasing systemic exposure during critical developmental windows [100]. Furthermore, desorption experiments demonstrate that tetracycline and Cd(II) desorb much more readily in simulated gastric fluid than in freshwater. This confirms that ingestion can trigger the release of contaminants even when environmental sorption appears stable [102]. This highlights the dynamic and reversible nature of microplastic–contaminant interactions within biological systems. Thus, these sorption mechanisms transform MNPLs into efficient carriers of metals, pharmaceuticals and PFAS, thereby increasing their toxicological significance during early life stages [96].

The sorption of PFAS is driven by a distinct set of mechanisms due to their amphiphilic structure and strong C-F bonds. Long-chain PFAS exhibit a high affinity for hydrophobic and moderately polar polymers, forming interactions through the hydrophobic partitioning of fluorinated tails and the electrostatic or hydrogen bonding of polar head groups. PET and PVC, which possess polar functional groups, demonstrate particularly strong PFAS sorption [100]. The weathering of MNPLs increases surface roughness and introduces oxygen-containing functional groups, thereby enhancing PFAS binding and persistence [20,22]. Once ingested, PFAS may desorb in the gastrointestinal tract, where bile salts and proteins compete for binding sites, thereby increasing systemic exposure during early development. This competitive binding environment is a key determinant of the internal dose and can substantially modify toxicokinetic profiles compared to environmental conditions [105]. Together, these sorption mechanisms transform MNPLs into efficient and dynamic carriers of metals, pharmaceuticals and PFAS, thereby increasing their toxicological significance during critical early life stages.

### 3.3. The Influence of Weathering, Oxidation and Biofilm Formation on Sorption Efficiency

A growing body of research shows that environmental weathering can substantially modify the ecological behavior, sorption dynamics, and toxicological relevance of MNPLs in terrestrial and aquatic systems. As emphasized by Liu et al. [106], environmental ageing significantly alters the physicochemical properties of microplastic surfaces, thereby increasing their capacity to adsorb and concentrate environmental contaminants. Pei et al. [107] observed a similar trend for mask-derived MNPLs, highlighting that even recently emerging plastic waste streams undergo rapid surface transformation under environmental exposure. Physical abrasion, UV radiation and mechanical fragmentation generate cracks, pits and irregular surface topographies, thereby increasing the available surface area and creating new sorption sites. According to Duan et al. [108], these processes introduce oxygen-containing functional groups (e.g., carbonyl, hydroxyl and carboxyl groups) and disrupt polymer crystallinity, thereby increasing chemical reactivity and sorption potential. UV-induced oxidation, as described by Wang et al. [109] and Xu et al. [110], increases surface polarity and negative charge density further, intensifying interactions with pharmaceuticals and metal ions. In parallel, weathering processes may facilitate the early colonization of biofilms, which further modifies surface chemistry and contaminant-binding behavior.

These structural and chemical changes enhance the retention of both hydrophobic and polar contaminants by providing micro-niches that facilitate partitioning, adsorption, and entrapment. As Shang et al. [111] demonstrated, weathered polyethylene in paddy soils becomes 1.1–2.2 times rougher and up to 3.6 times more oxidized. This markedly increases the sorption of cadmium and bisphenol A (BPA) via electrostatic attraction and ligand exchange, while simultaneously reducing the hydrophobic partitioning of di(2-ethylhexyl)phthalate (DEHP) due to increased surface hydrophilicity [111].

Comparative analyses by Ho et al. [112] further reveal that weathered polystyrene exhibits suppressed multilayer sorption of hydrophobic UV filters when hydrophobic cosolutes compete for binding sites and enhanced multilayer formation when hydrophilic cosolutes promote hydrogen bonding with oxidized surfaces. However, Wang et al. [109] report that aged polyethylene preferentially sorbs more hydrophobic pharmaceuticals, such as sertraline. This demonstrates that the type of polymer and its ageing history can strongly influence sorption hierarchies and contaminant selectivity. These contrasting findings emphasise that sorption behavior is highly context-dependent, influenced by environmental chemistry and polymer-specific ageing pathways.

Weathered particles exhibit increased surface roughness, promoting the adhesion of organic pollutants and metal ions via mechanical interlocking and enhanced van der Waals interactions. As emphasized by Pei et al. [107], the surfaces of chemically modified weathered MNPLs facilitate the binding of heavy metals, organic toxins, pesticides, antibiotics, and antibiotic-resistance genes via electrostatic interactions, precipitation processes, and microbial bioaccumulation. The development of biofilms can further strengthen these effects by introducing extracellular polymeric substances (EPS), which provide additional binding sites and alter surface hydrophobicity. Consequently, weathered MNPLs frequently demonstrate significantly higher sorption efficiency than pristine particles, making them more potent vectors of environmental contaminants in early-life exposure scenarios. Ecologically, Liu et al. [113] argue that these transformations increase the probability of ingestion and the risk of toxicity for aquatic organisms, while Ho et al. [112] demonstrate that multilayer pollutant coatings on weathered MNPLs can inhibit microalgal growth. This highlights the broader, ecosystem-level risks associated with aged plastic debris.

**Figure 2:**
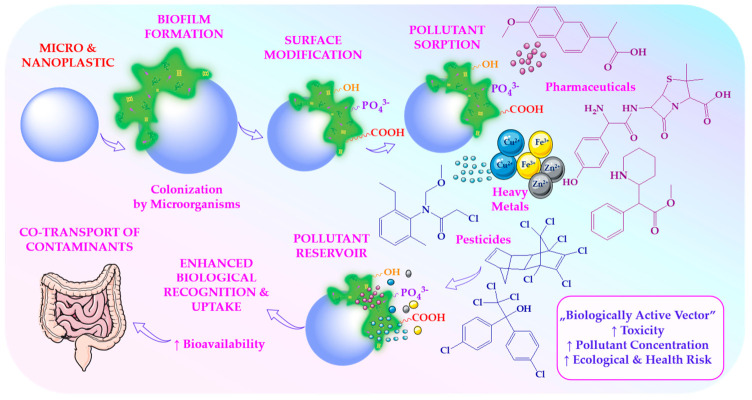
Biofilm formation on MNPLs as a driver of contaminant sorption and biological interactions. Biofilm formation on MNPLs creates a biologically active surface that alters their physicochemical properties and enhances the sorption of metals, pesticides, and pharmaceuticals through EPS functional groups. Biofilm-coated MNPLs show increased biological recognition and uptake, facilitate contaminant co-transport, and promote the spread of antibiotic resistance genes, thereby increasing ecological and potential human health risks through trophic transfer. Image provided by Servier Medical Art (https://smart.servier.com/), licensed under CC BY 4.0 (https://creativecommons.org/licenses/by/4.0/, accessed on 15 April 2026).

Figure 2 shows how the formation of biofilms on MNPLs modifies their surface properties, enhancing their ability to absorb and retain environmental contaminants such as metals, pesticides and pharmaceuticals. It also illustrates how biofilm-coated MNPLs promote biological uptake, contaminant co-transport, trophic transfer and the dissemination of antibiotic resistance genes, thereby posing increased risks to the environment and human health.

These studies demonstrate that environmental weathering is a key driver of microplastic reactivity. Physical abrasion, UV exposure and mechanical fragmentation progressively generate surface irregularities and introduce oxygen-containing functional groups. This increases sorption capacity and chemical reactivity, as described by Liu et al. [113] and Duan et al. [108]. These transformations create micro-niches that enhance the retention of hydrophobic and polar contaminants through processes such as hydrophobic partitioning, hydrogen bonding, electrostatic attraction and van der Waals forces, as further supported by Shang et al. [111]. As surfaces become increasingly rough and oxidized, weathered particles promote the stronger adhesion of pollutants and metal ions through mechanical interlocking and altered surface energetics. This is consistent with the observations of Pei et al. [107]. These processes explain why weathered MNPLs often behave as chemically active, environmentally persistent contaminant carriers rather than inert particles. Consequently, weathered MNPLs exhibit substantially higher sorption efficiency than pristine plastics, making them more effective vectors of environmental contaminants in early-life exposure scenarios, as highlighted by Ho et al. [112].

Oxidation-driven modifications and chemical reactivity are also key processes that govern the behavior of MNPLs, as environmental ageing progressively introduces oxygen-containing functional groups, such as carbonyls, hydroxyls, and carboxyls. The presence of these groups has been well documented in XPS analyses of UV-aged polyethylene and polystyrene by Di Giulio et al. [114]. Such surface oxidation modifies not only the chemical composition, but also the interfacial reactivity and contaminant-binding potential. These oxidative moieties increase surface polarity, generating negatively charged sites that can form strong electrostatic interactions and coordination complexes with metal ions, including Pb^2+^, Cd^2+^ and Cu^2+^. This mechanism is consistent with long-term weathering simulations reported by Yu et al. [115]. Oxidation also enhances the capacity for hydrogen bonding, thereby improving the sorption of polar pharmaceuticals and PFAS. This has been demonstrated in studies of the seawater interface of weathered polystyrene by Changfu et al. [116]. However, oxidative degradation weakens the polymer matrix, facilitating the release of additives and low-molecular-weight intermediates that modify surface chemistry further and alter contaminant affinity. This process is emphasized in Hu et al.’s [117] reviews of photoaging and AOP-induced ageing. Consequently, oxidation acts as both a surface-activating and a structure-degrading mechanism, dynamically reshaping the reactivity of MNPLs over time.

Reactive oxygen species (ROS), including ^•^OH and ^1^O_2_, which are generated during photooxidation, play a central role in these transformations. Cao et al. [118] have clearly demonstrated their contrasting effects on aromatic versus aliphatic polymers, highlighting polymer-dependent degradation pathways. Furthermore, dissolved organic matter derived from MNPLs introduces additional photoreactive components that undergo selective degradation depending on aromaticity and saturation, as revealed by high-resolution mass spectrometry analyses conducted by Chen et al. [119]. These processes create a feedback loop in which photochemical degradation products further enhance surface reactivity and contaminant-binding capacity. These oxidation-driven mechanisms transform MNPLs into chemically dynamic particles with a heightened affinity for diverse pollutants, thereby increasing their toxicological relevance to developing organisms.

As reported by Liu et al. [120], many studies have demonstrated that microbial colonization and biofilm formation can fundamentally alter the surfaces and environmental behavior of MNPLs. Biofilm formation introduces a biological conditioning layer consisting of microbial cells, EPS, proteins, and polysaccharides. This process creates dense microniches that support high microbial activity, as demonstrated by Wang et al. [121]. This biogenic layer modifies surface charge, hydrophobicity, and chemical reactivity, often increasing the affinity for organic and inorganic contaminants. This is consistent with observations in coastal seawater showing enhanced Cd(II) accumulation on biofilm-coated particles [122]. The EPS matrix provides abundant functional groups, such as carboxyl, phosphate and hydroxyl moieties, which bind metals, pesticides and pharmaceuticals with high specificity.

This mechanism is supported by evidence of strong heavy metal sorption on environmentally aged MNPLs, as reported by Gao et al. [123]. Importantly, biofilms also act as dynamic microreactors where microbial metabolism can transform sorbed contaminants, modifying their toxicity and persistence. Biofilms can trap contaminants within their matrix, creating a reservoir of pollutants that can be released into the gastrointestinal tract of developing organisms. Zhou et al. [124] emphasise this risk in ecological assessments of biofilm-mediated MNPLs. Notably, biofilm-conditioned MNPLs exhibit enhanced biological recognition and uptake, thereby increasing the likelihood of contaminant co-transport during early development. This biological conditioning also facilitates horizontal gene transfer processes, accelerating the spread of antibiotic resistance genes, as demonstrated by Zheng et al. [125]. These findings suggest that biofilm formation not only modifies microplastic surfaces, but also transforms them into biologically active and chemically reactive vectors. These particles can concentrate pollutants, mobilise metals and enhance microbial gene exchange processes, including the dissemination of antibiotic resistance. This significantly increases the ecological complexity and hazard potential of MNPLs compared to pristine particles. Consequently, this poses multifaceted risks to the early life stages of aquatic organisms, as well as to human health, via trophic transfer and environmental exposure pathways.

## 4. Formation of Microplastic–Contaminant Complexes and Their Stability in Biological Environments

### 4.1. Factors Controlling Microplastic–Contaminant Release

Understanding how MNPLs release sorbed contaminants under gastrointestinal (GI) conditions requires evidence from studies on surface chemistry, sorption kinetics, ionic interactions and biomolecular modification. These factors determine desorption timing and bioavailability. The strong pH-dependence of the surface charge of MNPLs, which is driven by the protonation and deprotonation of carboxylic, ester and hydroxyl groups, has been demonstrated for the six main polymer types: LDPE, HDPE, PP, PS, PVC and PET. This indicates that deprotonation at a pH greater than around 2 increases the negative surface charge and modifies the affinity of contaminants [126]. This behavior is consistent with the pH-dependent sorption of pharmaceuticals such as sulfamethoxazole, propranolol and sertraline, where shifts in ionization state between pH 2 and 12 modulate electrostatic interactions and hydrophobic partitioning on polyethylene MNPLs [109]. Previous studies on hydrophobic contaminants have also shown that polycyclic aromatic hydrocarbons (PAHs) strongly adsorb onto polystyrene nanoplastics through hydrophobic interactions. However, surface polarity changes induced by ageing progressively weaken these interactions and promote desorption [127]. Furthermore, non-linear sorption-desorption behavior and coupled mass transfer processes emphasise the significance of boundary conditions and sorption regimes, including the Henry, Langmuir, and Langmuir-Freundlich isotherms [128]. Diffusion modelling confirms that intraparticle diffusion coefficients decrease with decreasing particle size due to higher polymer crystallinity. This results in stronger contaminant retention and delayed release under dynamic gastrointestinal pH conditions [129].

The ionic strength has an equally important regulatory effect. Multivalent cations, such as Ca^2+^ and Mg^2+^, compete with sorbed metals for negatively charged sites on oxidized MNPLs. This displaces the metals through ligand-exchange mechanisms that are similar to those seen in microplastic–mineral aggregation systems. In these systems, Ca^2+^ bridging dramatically enhances attachment [130]. Elevated concentrations of NaCl and CaCl_2_ compress the electrical double layer, reducing the Debye length and weakening long-range electrostatic attraction. This is consistent with porous-media transport studies showing that ionic strength and mineralogy govern microplastic mobility and retention [131]. Importantly, ageing processes such as UV irradiation introduce oxygen-containing functional groups (–COOH and –OH), thereby increasing hydrophilicity and the negative surface charge. This enhances both contaminant sorption and subsequent ion-driven desorption [132].

Environmental macromolecules further modulate these interactions: humic acids sterically stabilise MNPLs and shift critical coagulation concentrations [88,133], whereas protein coronas alter particle size, surface chemistry, and aggregation kinetics through steric hindrance and molecular bridging. This reshapes desorption behavior, as demonstrated in multiple studies [134,135,136]. Additionally, biomolecular coatings act as dynamic interfaces that continuously exchange ligands with surrounding solutes, thereby increasing system variability and the unpredictability of contaminant release. Further research shows that the type and concentration of proteins strongly influence nanoplastic aggregation and surface reactivity, thereby reinforcing the central role of biomolecular coatings in environmental behavior [136].

Furthermore, these findings demonstrate that the release of contaminants from MNPLs is not governed by a single parameter, but by the combined effects of pH-driven protonation equilibria, electrolyte composition, polymer crystallinity, age-induced functionalization and biomolecular adsorption [126,128]. Studies showing that surface charge, ionic strength and sorption boundary conditions jointly regulate contaminant-polymer interactions support this conclusion [129,130]. These factors interact to determine bioavailability. For instance, research indicates that hydrophobic partitioning, electrostatic interactions, and macromolecule-mediated surface modification influence desorption behavior across different polymers and environmental conditions [132,135].

Importantly, these mechanisms do not act independently, but rather form a coupled physicochemical system in which changes in one parameter (e.g., pH or ionic strength) cascade through multiple interaction pathways. These mechanisms can substantially increase internal exposure in developing organisms, which have higher gastric pH, more permeable epithelial barriers and immature detoxification systems. This makes early life stages disproportionately vulnerable to contaminants associated with MNPLs. Recent biomedical analyses demonstrate that MNPLs can traverse epithelial barriers and enter the systemic circulation. Furthermore, biocorona composition can influence toxicity outcomes by altering cellular recognition and uptake pathways [137,138,139].

Studies have also shown that retention time determines desorption phases, namely that it governs how long MNPLs are exposed to gastrointestinal fluids. This directly influences the transition between fast and slow desorption phases, which is consistent with the shift from surface-controlled to diffusion-controlled release described in sorption models [128,129]. In this context, retention time should be understood as both a physical parameter and a biological exposure amplifier, as it determines the duration of contact between MNPLs and dynamic digestive conditions. Prolonged exposure to gastric acid results in continued protonation of polymer functional groups, weakening electrostatic interactions with cationic metals and increasing cumulative release. This is consistent with observations that protonation reduces the negative surface charge and destabilises metal–polymer complexes [126].

In the intestine, prolonged contact with bile salts and fatty acids facilitates the extraction of hydrophobic contaminants via micelle-mediated processes. This process is analogous to the hydrophobic partitioning of PAHs and pharmaceuticals into nonpolar phases, as observed for polystyrene and polyethylene MNPLs [127,133]. Longer retention also enables contaminants trapped in deeper polymer layers to diffuse outwards, shifting desorption from a surface-controlled regime to a diffusion-controlled regime described by Fickian kinetics. This is consistent with the finding that the intraparticle diffusion coefficient decreases with increasing polymer crystallinity and the depth to which the contaminant has penetrated [129]. This indicates that retention time increases total desorption by facilitating rapid surface release and slower diffusion-driven liberation from the polymer core. This dual-phase pattern is also evident in non-linear desorption behavior [128].

As demonstrated earlier, biocorona-mediated regulation of desorption is a significant influencing factor. The adsorption of proteins, lipids, polysaccharides, and microbial metabolites onto microplastic surfaces modifies surface chemistry and interaction energies. This is consistent with the observation that biocoronas fundamentally alter the behavior of MNPLs in biological systems [138]. Crucially, the biocorona acts as a dynamic and reversible interface that continuously reshapes the strength of contaminant binding depending on environmental and physiological conditions. Protein coronas can stabilise sorbed contaminants through additional hydrogen bonds, hydrophobic pockets or π–π interactions involving aromatic amino acids. This mechanism is supported by studies showing that protein coatings restructure microplastic surfaces, modulating aggregation and reactivity [130,134]. Conversely, the enzymatic degradation of the biocorona by proteases or lipases can expose fresh polymer surfaces and disrupt stabilizing interactions. This increases desorption rates and is analogous to the increased mobility and contaminant release observed after UV-induced surface renewal [134].

EPS produced by the gut microbiota contains carboxylate and phosphate groups that can exchange ligands with metal ions, thereby displacing them from the surface of MNPLs. This mechanism is similar to ligand-exchange interactions reported for environmental macromolecules [140]. At the same time, EPS layers can physically entrap contaminants within a hydrated, gel-like matrix, thereby delaying their release and creating secondary exposure reservoirs within the gut environment. Furthermore, EPS can sequester contaminants within the biofilm matrix, thereby altering their desorption pathways. This is consistent with the finding that macromolecular coatings modulate nanoparticle uptake, toxicity, and gene expression in human cells [141,142]. Furthermore, the composition of biocoronas has been shown to directly correlate with toxicity outcomes. For instance, the protein corona profiles of true-to-life MNPLs have been shown to predict intestinal epithelial responses [139]. Similarly, studies in marine invertebrates demonstrate that biocorona formation modulates immune responses and xenobiotic resistance, thus reinforcing the notion that the composition of the biocorona dictates biological interactions [137]. Therefore, depending on its composition, enzymatic turnover and biological context, the biocorona can stabilise or destabilise contaminant–polymer complexes. This conclusion is supported by studies on MNPLs [135,136,138].

### 4.2. Desorption Kinetics Under Varying pH Levels, Ionic Strengths and Enzymatic Activities

It is essential to understand how MNPLs release sorbed contaminants under gastrointestinal conditions in order to assess exposure risks, particularly in developing organisms whose digestive physiology, epithelial permeability and detoxification systems are not yet fully mature. Studies on sorption-desorption mechanisms provide converging evidence that pH, ionic strength, and polymer surface chemistry jointly determine when contaminants become bioavailable by detaching from MNPLs—a process that may disproportionately affect infants and juveniles [126,128,133]. Also, enzymatic activity (e.g., esterases, proteases, and bile-associated lipases) can accelerate the degradation of surface-bound complexes, particularly during the intestinal phase of digestion. This modulates the release kinetics, affecting not only physicochemical processes, but also biological ones.

The desorption kinetics of microplastic–contaminant complexes are fundamentally controlled by protonation-deprotonation equilibria that change throughout the gastrointestinal tract. These equilibria alter the surface charge and interaction energies in a manner consistent with the pH-dependent surface acidity profiles observed in multiple polymer types [126,133]. In the acidic stomach, the protonation of carboxyl, hydroxyl and carbonyl groups on weathered MNPLs decreases their negative surface charge. This weakens the electrostatic attraction to cationic metals and accelerates their release. This mechanism is consistent with the observation that protonation suppresses anionic surface sites and enhances contaminant mobility [126]. Pharmaceuticals containing amine groups undergo increased protonation at low pH, enhancing solubility and reducing affinity for hydrophobic polymer domains. This mirrors the pH-dependent sorption behavior of sulfamethoxazole, propranolol and sertraline on polyethylene MNPLs, where the ionization state strongly dictates the sorption affinity [133].

By contrast, hydrophobic pesticides and PFAS are more strongly associated with MNPLs under gastric conditions and persist until reaching the small intestine. There, changes in pH and bile composition modify the polarity of the polymer surface and reduce hydrophobic partitioning. These findings are consistent with the nonlinear sorption-desorption dynamics and hydrophobic interactions that have been reported for organic contaminants on polystyrene and polyethylene [128,132]. Diffusion models further support these patterns by demonstrating that the timing of desorption is determined by a combination of sorption boundary conditions, polymer crystallinity and intraparticle diffusion coefficients. Smaller, more crystalline particles exhibit slower internal diffusion and altered release profiles [129]. These findings demonstrate that pH-dependent protonation states directly regulate the strength and timing of desorption events.

Ionic strength is also a key determinant of contaminant release. Studies have shown that ion-exchange reactions, charge screening and competitive binding together control desorption behavior [130,131]. In intestinal fluids enriched in Na^+^, K^+^ and Ca^2+^, multivalent cations can outcompete sorbed metals for negatively charged sites on oxidized MNPLs, displacing them through ligand-exchange mechanisms analogous to those observed in Ca^2+^-mediated bridging in microplastic-mineral aggregation systems [130]. Increasing ionic strength compresses the electrical double layer and reduces the Debye length. This diminishes long-range electrostatic attraction and destabilizes complexes with polar pharmaceuticals or PFAS. This is consistent with the mobility changes reported for aged and functionalized MNPLs in porous media [131,134]. Chloride and bicarbonate ions can also form outer-sphere complexes with metals, thereby increasing their solubility and accelerating desorption.

This process is analogous to the competitive sorption effects observed in multi-solute pharmaceutical systems, wherein hydrophobic compounds such as sertraline occupy sorption sites preferentially, displacing other molecules [133]. These processes are further modulated by environmental macromolecules. Protein coronas, humic substances, and biomolecular coatings alter surface charge, steric interactions, and binding equilibria, thereby reshaping aggregation and desorption behavior, as demonstrated in several studies [134,135,136]. The evidence suggests that ionic composition acts as a molecular lever that weakens sorption forces and promotes contaminant release.

These processes are particularly important for developing organisms, whose gastrointestinal physiology differs markedly from that of adults. Infants and juveniles have a higher gastric pH level, greater epithelial permeability, immature detoxification pathways, reduced enzymatic efficiency and a rapidly evolving microbiome. These factors can increase pH- and ion-driven desorption processes, thereby raising systemic exposure to metals, pharmaceuticals and PFAS released from MNPLs [143]. Furthermore, the prolonged residence time of particles in the intestine in early life increases the window for desorption and uptake, thereby enhancing the internal dose even at low environmental exposure levels [144]. Therefore, it is essential to understand how pH and ionic strength regulate desorption in order to predict exposure risks in early life and assess the unique vulnerability of developing organisms to contaminants associated with MNPLs.

### 4.3. Competitive Binding Between Contaminants and Natural Organic Matter (NOM)

Natural organic matter (NOM) can significantly impact the behavior of contaminants associated with MNPLs. Multiple studies have demonstrated that NOM acts as a potent molecular competitor, capable of releasing pollutants that were previously stabilized on polymer surfaces. Song et al. [145] demonstrated that dissolved organic matter (DOM) contains an abundance of aromatic rings, carboxylates, phenols, and hydrophobic domains that actively engage in hydrogen bonding, π–π interactions, and multidentate metal coordination. This confirms that NOM possesses the precise functional groups necessary to outcompete oxidized microplastic surfaces for pesticides, pharmaceuticals, PFAS, and metal ions. In practice, NOM acts as a dynamic ligand reservoir in aquatic and biological systems, continuously exchanging binding partners with pollutants and polymer surfaces. This behavior is consistent with the findings of Ding et al. [146], who demonstrated that polystyrene MNPLs hetero-associate with diverse DOM types, and that DOM can displace sorbed contaminants by forming more stable aromatic and carboxylate-driven complexes. Regarding metal ions, Lee and Hur [147] provide further support for the competitive advantage of NOM by showing that natural DOM forms stronger copper-binding complexes than microplastic-derived DOM. This indicates that NOM can readily strip metals from polymer surfaces through bidentate or multidentate coordination. Yan et al. [148] also reinforce the idea of hydrophobic contaminant mobilization, finding that both MP-DOM and natural DOM bind pharmaceuticals through hydrophobic partitioning and π–π stacking. Natural DOM often exhibits a higher affinity due to its structural complexity. Complementary evidence from Choi et al. [149] showed that the photodegradation of MNPLs produces increasingly reactive DOM that interacts synergistically with natural NOM to enhance the solubilization of contaminants.

At the ecosystem level, Sheridan et al. [150] showed that plastic-derived NOM is more bioavailable than lake NOM and stimulates microbial metabolism more strongly. This highlights that NOM-plastic interactions are chemical and biological, influencing the transformation of contaminants downstream. This suggests that NOM can act as both a competitor and a driver of transformation, reshaping the speciation of contaminants while simultaneously enhancing the microbial processing of plastic-derived compounds. Further analytical studies reveal the extent of the interaction between NOM and MNPLs. For example, Lee et al. [151] developed fluorescence indicators that can distinguish MP-DOM from NOM. Meanwhile, Le Juge et al. [152] and Zhang et al. [153] demonstrated that NOM can complicate the detection of MNPLs due to the overlap of their pyrolysis and UV-Vis signatures. This underscores the intimate molecular association between plastics and NOM. Finally, Tao et al. [154] showed that the migration of polystyrene particles on natural substrates is jointly mediated by NOM and microbial biofilms. This reinforces the idea that MNPLs and their associated contaminants are physically redistributed within environmental matrices as well as being chemically competed for by NOM.

Furthermore, these studies reveal that, when NOM encounters microplastic–contaminant complexes in gastrointestinal fluids, its functional groups can displace metals, pesticides, pharmaceuticals, and PFAS from polymer surfaces. This process transforms surface-bound contaminants into soluble, mobile forms because NOM–contaminant complexes are more thermodynamically stable than polymer–contaminant interactions, especially under biologically relevant ionic and pH conditions. This transformation is highly relevant to developing organisms, whose gastrointestinal environments are characterized by looser epithelial junctions, greater membrane permeability, reduced detoxification capacity and different mucus chemistry [140,144]. Under these conditions, it is important to note that NOM-mobilized contaminants diffuse more readily, interact more efficiently with micellar carriers, and cross epithelial barriers with fewer physiological constraints. This amplifies systemic exposure and increases the toxicological impact of MNPLs during early life.

There is strong support from multiple lines of evidence for the increased bioaccessibility of pollutants during early development. This shows that MNPLs act as dynamic platforms whose contaminant load can be rapidly reconfigured by NOM and biofilm-associated processes, rather than as inert carriers. Studies by Pan et al. [155] on biofilm-coated MNPLs demonstrated that microbial colonization fundamentally alters polymer surface chemistry, introducing enzymatically active and ligand-rich microenvironments that significantly increase sorption heterogeneity and desorption variability. This enhances the adsorption and subsequent release of heavy metals in gastrointestinal environments, with direct implications for neurotoxic outcomes such as oxidative stress, mitochondrial dysfunction, and metal dysbiosis. These findings are consistent with those of Wu et al. [156], who demonstrated that smaller microplastic particles accumulate denser biofilms and consequently carry higher metal loads. This increases the likelihood that these metals will become bioaccessible when exposed to digestive fluids. Additionally, the scaling effect created by particle size-dependent biofilm density means that nanoscale plastics act as disproportionately efficient contaminant vectors.

This vulnerability is also evident in wider studies on contaminant transfer. For instance, Yang et al. [157] documented the efficient migration of trace metals through soil-plant-human systems, showing that metal mobility increases when they are bound to organic matrices. Meanwhile, Zhou et al. [158] revealed that MNPLs intensify cadmium toxicity in earthworms by boosting metal absorption and physiological stress. These findings resemble the behavior of NOM in the developing gut, where mucins, dietary lipids and microbial metabolites act as natural organic matter with a strong binding capacity. In this biological context, NOM functions not only as a competitor, but also as a chemical mediator that continuously reshapes contaminant partitioning equilibria. When NOM encounters microplastic–contaminant complexes, it can extract hydrophobic pesticides, PFAS and metals from polymer surfaces, thereby increasing their solubility and shifting them into highly bioaccessible forms [153,154].

This is consistent with the early bioaccessibility models of Brandon et al. [159] and Yu et al. [160], which demonstrated that contaminants bound to particulate matter become significantly more bioavailable when exposed to gastrointestinal fluids rich in organic ligands. These effects are amplified during development, when early-life epithelial junctions are looser, detoxification systems are immature, and mucus composition is less structured, thereby favoring the diffusion and uptake of NOM-bound contaminants. Consequently, exposure increases in both magnitude and duration due to slower clearance and higher intestinal permeability.

Additional evidence from environmental exposure studies reinforces this vulnerability. Kowalczyk et al. [161] identified polyethylene packaging as a significant source of MNPLs entering food systems. Meanwhile, Voets et al. [162] and Wolinski et al. [163] demonstrated that high microplastic exposure compromises the physiological condition of aquatic organisms and increases contaminant accumulation, thus highlighting that MNPLs consistently enhance the bioavailability of pollutants across taxa. Thus, these studies reveal that competitive binding by NOM transforms MNPLs from passive reservoirs of pollutants into active catalysts of contaminant mobilization, dramatically increasing exposure during critical periods of development when biological defenses are least mature.

### 4.4. Stability of Complexes in the Gastrointestinal Fluids of Developing Organisms

Understanding how microplastic–contaminant complexes behave in the gastrointestinal tract of developing fish is essential to explain their heightened sensitivity to exposure to a mixture of pollutants during the early stages of life [164]. When these complexes enter an immature digestive system, they encounter a substantially different biochemical environment than that of adults, including reduced gastric acidity, lower enzymatic activity, and an incompletely developed bile composition [165,166]. Additionally, the intestinal barrier is more permeable in early life, potentially facilitating the translocation of both free contaminants and particle-bound forms into the systemic circulation. These physiological conditions can stabilize microplastic–pollutant associations by slowing chemical breakdown and reducing competitive displacement processes [167]. Reduced enzymatic activity may also delay the degradation of protein-rich biocoronas, enabling contaminants to remain bound to MNPLs for longer and prolonging their residence time in the developing gastrointestinal tract [166]. This prolonged retention increases the probability of chronic internal exposure during critical windows of organogenesis and tissue differentiation.

As these complexes pass from the stomach to the intestine, changes in pH and the presence of bile salts become important factors in determining their stability and how contaminants are released. Acidic gastric conditions can weaken metal–polymer interactions, promoting the release of cationic species. In contrast, the neutral to slightly alkaline intestinal environment favors the desorption of hydrophobic pesticides and pharmaceuticals [168]. Meanwhile, due to their amphiphilic structure, bile salts can compete with MNPLs for the binding of hydrophobic and polar contaminants, such as PFAS, effectively displacing them from particle surfaces [167]. This competitive binding mechanism is particularly relevant during early development when bile composition and secretion rates are still immature and highly variable. Together, these processes demonstrate how pH gradients, enzymatic activity, and bile composition act as natural chemical switches that regulate the release of contaminants from MNPLs during gastrointestinal transit.

As summarized in Table 2, which details the effects of MNPLs on fish development, including co-exposure mechanisms and transgenerational impacts, the findings from developmental and mixture toxicity studies suggest that MNPLs can interfere with early physiological processes and interact with concurrent chemical contaminants, increasing toxicity at various biological levels. These interactions extend beyond acute toxicity and may result in long-term consequences, including altered gene expression, metabolic reprogramming, and reproductive impairment. This framework helps us to understand how particle–pollutant complexes behave within the immature gastrointestinal environment described above and highlights the importance of considering the chemical and physiological contexts when assessing the risks of exposure in early life.

A growing body of research has demonstrated that developing aquatic organisms are uniquely vulnerable to the toxic effects of MNPLs. A broad scientific consensus has emerged from multiple studies that integrate exposure, uptake and developmental outcomes across taxa [164]. In particular, MNPLs have been shown to penetrate embryonic barriers with remarkable ease and accumulate in sensitive tissues. For example, Pitt et al. [165] documented the presence of polystyrene MNPLs in the yolk sac, gastrointestinal tract and brain of zebrafish embryos, revealing that even the earliest developmental stages lack effective physiological barriers against particle uptake. In contrast, Teng et al. [166] demonstrated that long-term exposure to 44 nm polystyrene nanoparticles disrupts the brain–gut–microbiota axis, leading to stunted growth, delayed hatching, and impaired locomotion. These findings complement each other: Pitt et al. [165] show where particles go, while Teng et al. [166] show what they do once they get there.

Studies by Teng et al. [166] have demonstrated that charge-dependent toxicity adds another layer of complexity. They showed that positively charged MNPLs induce markedly stronger neurobehavioral and cardiac defects than negatively charged particles. This highlights how surface charge modulates developmental toxicity. However, this is in contrast to the broader patterns described by Huang et al. [164], who emphasize oxidative stress, apoptosis and immune disruption as common pathways across multiple studies, regardless of particle charge. Alongside the insights of Sofield et al. [174], who demonstrate that MNPLs disrupt gut integrity and interfere with gut-brain signaling, these findings reveal that the interaction between particle chemistry and physiological immaturity amplifies harm.

Notably, when contaminants desorb from MNPLs, they encounter an epithelial barrier that is still developing and is characterized by looser tight junctions, thinner mucus layers, and reduced expression of detoxification enzymes [175,176]. These features substantially increase intestinal permeability, allowing desorbed toxicants to enter the systemic circulation more readily than in adults. Hydrophobic compounds may partition directly into enterocyte membranes, while polar pharmaceuticals and PFAS can exploit transporter proteins or paracellular pathways [177]. The presence of MNPLs can exacerbate this process by inducing mild inflammation or oxidative stress, further compromising barrier integrity [174]. These studies emphasize that desorption does not occur in isolation, but rather interacts with the physiological vulnerabilities of early life stages to enhance contaminant uptake and intensify developmental toxicity.

The biological components of the developing gut introduce additional mechanisms that increase risk. For example, mucus glycoproteins can temporarily shield MNPLs, stabilizing some complexes while displacing others. Early-life microbiota are less diverse but produce metabolites that can modify particle surfaces and weaken hydrogen bonds or electrostatic interactions [164]. Furthermore, the developmental immaturity of immune recognition systems reduces the ability to efficiently clear particle-contaminant complexes, while the slower intestinal transit of young organisms increases the residence time of MNPLs and creates more opportunities for desorption events. Thus, biological processes within the developing gut actively influence the bioavailability of contaminants.

As MNPLs -contaminant complexes progress through the gastrointestinal tract of developing organisms, desorption becomes the main process determining internal exposure. The acidic environment of the stomach can weaken the bonds holding metals and oxidized polymer surfaces together, thereby promoting their release into gastric fluid [168]. In contrast, hydrophobic pesticides and pharmaceuticals tend to remain tightly bound to MNPLs until they reach the intestine, where bile salts and digestive lipids act as powerful solubilizing agents. These amphiphilic molecules compete for hydrophobic binding sites, effectively displacing contaminants from the polymer matrix [167]. This illustrates the pivotal role of the gastrointestinal tract’s chemical composition in determining the timing of contaminant detachment from MNPLs and their subsequent availability for absorption.

Co-exposure studies provide some of the strongest evidence that plastics act as toxic amplifiers. For instance, Wang et al. [133] showed that PS-NPs greatly increased the developmental toxicity of BDE-47 in zebrafish embryos, worsening oedema, mortality, and thyroid disruption. Similarly, Shi et al. [170] found that MNPLs enhance the embryotoxicity of BDE-47 in the species *Hexagrammos otakii*, reducing hatchability and altering Wnt signaling. These results are consistent with an earlier observation by González-Doncel et al. [178], who found that exposure to waterborne PBDEs alone often underestimates true toxicity due to poor solubility and limited bioavailability. This highlights why plastics serve as potent carriers that effectively increase exposure to otherwise poorly accessible, hydrophobic contaminants.

Other contaminants follow the same pattern. For instance, Kandaswamy et al. [172] showed that nanoplastics intensify diclofenac-induced oxidative stress and intestinal damage. Furthermore, Kim et al. [171] revealed that combinations of MNPLs with BPS and MEHP result in malformations at non-toxic doses of the individual components. Further research by Aliakbarzadeh et al. [169] suggests that nanoplastics may increase the neurotoxicity of nonylphenol by disrupting neurotransmission and antioxidant defenses. Xia et al. [173] observed that MNPLs can increase the uptake and cytotoxicity of BDE-209 in invertebrates, supporting the view that plastics may enhance the bioavailability of contaminants across taxa. These co-exposure-related effects are often associated with increased oxidative stress, mitochondrial dysfunction and impaired detoxification capacity, suggesting a convergence on shared molecular stress pathways across chemically distinct pollutants.

Baseline polybrominated diphenyl ether (PBDE) studies provide important context. Mhadhbi et al. [179] demonstrated that fish embryos are inherently sensitive to BDEs 47 and 99, exhibiting lethality and malformations even in the absence of plastics. When these findings are considered alongside the co-exposure studies mentioned above, it becomes clear that MNPLs do not merely add to existing toxicity; they multiply it by increasing the internal dose, altering tissue distribution and modifying the bioavailability of contaminants at critical developmental stages.

Thus, a consistent pattern emerges across all authors and experimental systems: plastics prolong the residence time of contaminants in the developing gut; facilitate desorption at critical physiological transitions; disrupt endocrine and neural pathways; and increase the toxicity of hydrophobic pollutants [180]. Developing organisms with immature digestive, immune, and metabolic systems are therefore the most vulnerable. The evidence leaves little doubt that MNPLs act as potent toxic vectors that fundamentally reshape exposure to environmental contaminants in early life, transforming otherwise low-bioavailability chemicals into biologically active developmental stressors.

### 4.5. Toxicological Consequences During Development

Recent literature increasingly shows that MNPLs pose a disproportionate threat to developing organisms. Different authors have reached this conclusion from various complementary perspectives. For instance, Sofield et al. [174] emphasize that MNPLs can disrupt gut homeostasis and interfere with the gut-brain axis. This pathway is still developing during the early stages of life and is therefore highly susceptible to disruption. Their findings align with those of Dzierżyński et al. [177], who argue that MNPLs can accumulate in tissues, alter cellular signaling, and contribute to long-term pathological outcomes, including carcinogenesis. While their review focuses on humans, the described mechanisms—oxidative stress, chronic inflammation, endocrine disruption and impaired barrier integrity—closely mirror those observed in fish embryos and larvae. This indicates a strong conservation of toxicity pathways across species.

From an ecological and biological standpoint, Dong et al. [176] provide compelling evidence that MNPLs can translocate across biological barriers in a wide range of animals, including during early developmental stages. They emphasize that the immaturity of epithelial tissues, loose intercellular junctions and underdeveloped detoxification systems make embryos particularly permeable to plastic particles. This is supported by Ma et al. [175], who demonstrate that MNPLs can penetrate fish embryo blastopores, enter the circulation, and reach the tissues of the next generation. Their review also shows that MNPLs adhere to the surface of embryos and larvae, entering internal organs via ingestion or epidermal infiltration—pathways that are significantly more accessible during the early life stages than in adulthood.

The biological components of the developing gut introduce an additional layer of complexity. Mucus glycoproteins can temporarily shield MNPLs, stabilizing some contaminant complexes while displacing others. Early-life microbiota are less diverse but produce metabolites that can modify particle surfaces and weaken hydrogen bonds or electrostatic interactions [174,176]. Additionally, immature immune surveillance and reduced epithelial turnover limit the clearance of particle-associated contaminants further. Young organisms also exhibit slower intestinal transit, which increases the residence time of MNPLs and provides more opportunity for desorption and epithelial interaction [175]. Meanwhile, MNPLs may act as temporary reservoirs, releasing contaminants gradually rather than all at once, thereby prolonging exposure even after ingestion has ceased [177]. Thus, MNPLs can transform a brief exposure event into a sustained internal dose, increasing toxicological consequences during development.

As MNPLs -contaminant complexes progress through the gastrointestinal tract, desorption becomes the key process determining internal exposure. The acidic environment of the stomach can weaken the bonds between metals and oxidized polymer surfaces, thereby promoting their release into gastric fluid [176]. Conversely, hydrophobic pesticides and pharmaceuticals tend to remain tightly bound to MNPLs until they reach the intestine. There, bile salts and digestive lipids act as powerful solubilizing agents that compete for hydrophobic binding sites and displace contaminants from the polymer matrix [175]. These processes demonstrate that the gastrointestinal tract’s chemical composition plays a decisive role in determining the timing of contaminant detachment from MNPLs and their subsequent bioavailability.

Thus, the sequence of events governing the stability, desorption and bioavailability of microplastic-associated contaminants in the developing gastrointestinal tract is highly interconnected and context-dependent [9,12]. The immature biochemical environment stabilizes some complexes while destabilizing others, resulting in exposure patterns that are unique to early development and not observed in adults [10,32]. Specifically, pH transitions, bile composition, mucus interactions, microbial activity and epithelial immaturity determine the efficiency and timing with which contaminants detach from MNPLs and cross the intestinal barrier [9,12,143]. In summary, MNPLs transport contaminants into the gut and actively reshape the timing, intensity and biological impact of exposure during critical developmental periods. This makes early life stages the most vulnerable to plastic-associated toxicity [10,32,52].

## 5. Intestinal Uptake Mechanisms in Developing Organisms

### 5.1. Major Endocytic Pathways: Clathrin, Caveolin and Macropinocytosis

Understanding clathrin- and caveolin-mediated endocytosis, as well as alternative uptake routes, is crucial for evaluating the interaction of magnetic nanoparticle (MNP) systems with human cells. This is because these pathways determine whether biocorona-coated particles can cross epithelial barriers and initiate downstream biological effects [157]. Uptake efficiency is not solely determined by particle size, but also by the physicochemical identity of the biocorona, which acts as a dynamic “biological interface” between MNPs and cellular receptors.

Clathrin-mediated endocytosis (CME) is the most organized and receptor-driven internalization process. Its relevance to MNPL stems from the ability of biocorona proteins to mimic endogenous ligands, enabling particles to engage adaptor complexes such as AP-2 [181]. This adaptor-mediated recognition process is analogous to viral entry, where surface motifs dictate CME uptake selectivity. This illustrates how the composition of the biocorona can redirect particle trafficking [182]. Following adaptor recruitment, clathrin triskelions assemble into a curved lattice that drives membrane invagination. This process is tightly regulated by cytoskeletal dynamics and endocytic checkpoints [181,183]. Dynamin then constricts the vesicle neck and catalyses membrane scission through GTP hydrolysis, completing a pathway whose efficiency depends on regulatory proteins and post-translational modifications. Notably, these regulatory steps can be modulated indirectly by oxidative stress and membrane lipid alterations induced by MNPLs exposure. As CME preferentially internalizes nanoscale cargo that interfaces with the receptor–adaptor machinery, MNPLs with specific corona signatures can exploit this selectivity to enter epithelial or immune cells [158].

In parallel, caveolin-mediated endocytosis provides a second major uptake route for MNPLs. This route relies on cholesterol- and sphingolipid-rich membrane microdomains rather than clathrin scaffolds. Caveolae are stabilized by caveolin-1 and recruit cargo that either partitions into lipid rafts or interacts with raft-associated proteins. These behaviors are strongly modulated by the amphiphilic and protein components of the biocorona [151,153]. Unlike CME, caveolar uptake is highly sensitive to membrane composition, cholesterol availability, and cytoskeletal tension [183]. As biocorona formation alters the hydrophobicity, charge distribution and protein identity of particles, it can direct MNPs towards either CME or caveolar pathways. This determines their intracellular fate, trafficking efficiency and potential toxicity [157,158]. This pathway selectivity is important because caveolae are abundant in endothelial and epithelial tissues, which are key interfaces for systemic translocation. These findings demonstrate that the selection of the endocytic pathway is not incidental, but rather a central determinant of how MNPLs interact with human tissues.

Macropinocytosis is a fundamentally different third uptake route characterized by the non-selective engulfment of extracellular fluid through actin-driven membrane ruffling. Zhang et al. [184] have provided a detailed characterization of this mechanism, demonstrating that cancer-associated fibroblasts rely on PI3K-, Rac1- and Pak1-dependent signaling to internalize large extracellular structures. These findings align with those of García-Bermúdez et al. [185], who revealed that metabolically stressed cells increase macropinocytosis to enhance nutrient absorption. This confirms that the pathway is dynamically activated under conditions of cellular stress or high metabolic demand. Xu et al. [186] provided direct toxicological evidence that polystyrene MNPLs enter intestinal epithelial Caco-2 cells via macropinocytosis, particularly when particles are aggregated or surface-modified (e.g., PS-NH_2_, PS-COOH). This pathway efficiently internalizes particles that are too large or irregular for clathrin- or caveolin-mediated uptake. This is particularly pertinent in the context of environmentally weathered MNPLs, which frequently form hetero-aggregates with biomolecules and inorganic particles. Together, these studies demonstrate that macropinocytosis acts as an efficient entry route for MNPLs. This is particularly concerning for developing organisms due to the elevated levels of actin dynamics, membrane turnover, and epithelial permeability that occur during growth [186].

In addition to these classical mechanisms, cells utilize clathrin- and caveolin-independent endocytic pathways, referred to as CLIC/GEEC routes. These pathways are regulated by small GTPases, such as Cdc42, Arf6 and RhoA, which control membrane curvature and cytoskeletal tension. This was highlighted by Gerasymchuk et al. [183] in their analysis of microRNA-mediated regulation of endocytosis. These findings complement those of Lamoree et al. [157], who demonstrated that the biocorona formed on magnetic nanoparticles (MNPs) determines interactions with membrane lipids, including glycosphingolipids and phosphoinositides. These lipids are key determinants of CLIC/GEEC uptake. Furthermore, Brouwer et al. [158] showed that true-to-life MNPs acquire distinct protein corona signatures that correlate with epithelial toxicity and cytokine secretion. This indicates that corona-dependent lipid interactions directly influence both uptake and immune responses. Thus, CLIC/GEEC pathways provide an additional entry route for MNPs whose biocorona favors lipid-driven internalization.

It is important to note that in developing organisms, membrane lipid composition, GTPase signaling networks and epithelial barrier maturation are still evolving. This developmental immaturity increases membrane plasticity and endocytic activity, potentially increasing internalization rates of particles across all pathways. Consequently, clathrin-, caveolin-, macropinocytosis- and CLIC/GEEC-mediated uptake may disproportionately dominate during the early stages of life, thereby increasing internal exposure to micro- and nanoplastics and their associated contaminants [157,158].

Studies have emphasized that another important uptake mechanism is phagocytosis, which is a receptor-driven process usually carried out by professional phagocytes, such as macrophages and dendritic cells. In this process, binding of a ligand to Fc, complement, or scavenger receptors triggers an extension of the cell membrane around the particle via an actin-dependent mechanism, as demonstrated in various studies [187]. This mechanism is fundamentally linked to innate immune surveillance and is highly sensitive to the physicochemical properties of the particle surface.

This canonical description is directly relevant to MNPLs, as polystyrene particles acquiring a protein corona containing immunoglobulins or complement factors can be recognized as ‘foreign bodies’ and engulfed by microglia. This can lead to immune activation, inflammatory signaling and apoptosis in both in vitro and in vivo systems [188]. Consistent with this, exposure of pregnant females to polystyrene MNPLs has been shown to induce neurotoxicity and impair social behavior in offspring. This suggests that phagocytic uptake of plastic particles in the developing brain can result in long-term functional consequences [189]. Importantly, this implies that the biocorona not only regulates passive uptake pathways, but can also actively convert MNPLs into immunologically recognisable structures. Taken together, these findings imply that once MNPLs acquire a protein corona that mimics immunogenic or apoptotic surfaces, phagocytosis becomes a critical entry and clearance route, particularly in developing organisms, where microglia and other phagocytes actively sculpt neural and immune circuits [187,188,189].

In addition to phagocytosis, another endocytic route is flotillin-mediated endocytosis, which relies on flotillin-1 and flotillin-2 microdomains to organize cholesterol-rich membrane platforms. Recent work by Lu et al. [190] has demonstrated that flotillin complexes nucleate stable nanoclusters within lipid rafts. These nanoclusters form scaffolds that are capable of internalizing specific cargo via raft-associated proteins and lipids. Although this pathway has traditionally been studied in signaling and membrane organization, its biophysical characteristics—cholesterol dependence, raft partitioning, and nanocluster formation—make it highly relevant for nanosized particles whose surface chemistry or biocorona promotes lipid raft association [190]. In this context, flotillin-mediated uptake can be considered part of a broader family of lipid raft-dependent internalization routes that functionally overlap with caveolin-mediated endocytosis.

Taking a comparative approach, experimental methods such as RNA interference and nanoparticle tracking have revealed that non-clathrin-dependent uptake mechanisms can be selectively modulated, thus confirming the adaptability of these pathways [191]. Similarly, MNPLs with amphiphilic surfaces or protein-rich coronas can also localize to flotillin-positive membrane domains. This suggests that flotillin-mediated uptake is not restricted to endogenous signaling complexes, but can also be exploited by environmental particles, depending on the composition of their surface corona. This represents another lipid-driven mechanism that is particularly relevant in developing tissues, where membrane composition, raft organization, and cholesterol distribution are subject to continuous remodelling over time [157,190].

Furthermore, phagocytosis and flotillin-mediated endocytosis complement macropinocytosis and other uptake routes, demonstrating that cells possess multiple molecular entry mechanisms that are partially redundant and tuned to different particle properties, such as size, surface chemistry, biocorona composition, and membrane affinity [187,190]. Studies on the toxicology of polystyrene MNPLs demonstrate that particles can exploit multiple pathways simultaneously, including macropinocytosis and clathrin-mediated endocytosis in intestinal epithelial cells. This results in systemic distribution and barrier disruption in mice [186]. This multi-pathway redundancy increases the likelihood that at least one uptake route will be available under physiological conditions.

**Figure 3:**
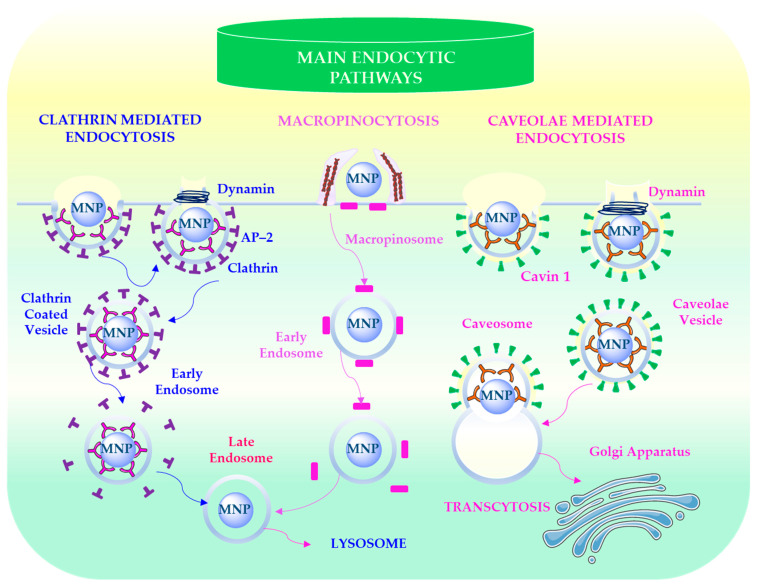
Major endocytic pathways mediating micro- and nanoplastic uptake: clathrin-dependent, caveolin-dependent, and micropinocytosis. Micro- and nanoplastics (MNP), particularly those coated with a biocorona, enter cells via clathrin-mediated endocytosis, caveolin-mediated endocytosis, and macropinocytosis. Clathrin-mediated uptake is receptor-dependent and selective, caveolin-mediated uptake occurs through lipid-rich membrane domains, while macropinocytosis enables non-selective internalization of larger or aggregated particles. MNP physicochemical properties and biocorona composition influence pathway selection, thereby determining cellular uptake, intracellular trafficking, and toxicological responses, particularly in developing tissues. Image provided by Servier Medical Art (https://smart.servier.com/), licensed under CC BY 4.0 (https://creativecommons.org/licenses/by/4.0/, accessed on 15 April 2026). Abbreviations: AP-2–adaptor protein complex 2; MNP–micro- and nanoplastics.

Figure 3 shows the main endocytic pathways involved in the cellular uptake of MNPLs. These pathways include clathrin-mediated endocytosis, caveolin-mediated endocytosis and macropinocytosis. It illustrates how the physicochemical properties and biocorona composition of MNPLs affect their internalization by cells, their intracellular trafficking and their toxicological effects, particularly in developing tissues.

Integrating these insights with evidence of immune activation, neurotoxicity, and behavioral alterations following MNPLs exposure [188,189] reveals that the diversity of entry routes significantly increases the likelihood of microplastic–contaminant complexes being successfully internalized by biological systems. This is particularly critical during early development when epithelial barriers, neural circuits, and immune surveillance systems are still maturing; the cytoskeleton is highly dynamic; and membrane receptors and lipid rafts are continuously reorganizing. Consequently, developing organisms exhibit a structurally and functionally ‘permissive’ cellular environment that favors particle uptake across multiple pathways simultaneously. This renders early life stages disproportionately vulnerable to plastic-associated exposures [157,158,192].

### 5.2. Paracellular Transport and Tight Junctions Disruption

Paracellular transport becomes increasingly prominent when the epithelial barrier undergoes molecular and structural rearrangements that weaken its sealing capacity. This process has been described in detail by Keaney and Campbell [193] in both epithelial and endothelial systems. The integrity of tight junctions normally depends on the coordinated assembly of claudin-1, occludin and ZO-1. The structural organization and cytoskeletal anchoring of these proteins have been characterized in detail by Yuki et al. [194]. The findings from renal epithelia, such as those reported by Muto [195], show that claudin composition determines segment-specific permeability and ion selectivity. While these studies do not examine the developing gut directly, they provide indirect insight into how the composition of tight junctions influences barrier behavior.

Inflammatory mediators and oxidative stress can alter the phosphorylation state of junctional proteins, thereby disrupting their conformational stability and membrane localization. As demonstrated by Cui et al. [196] and Hernández et al. [197], this process has also been reported in MNPL-induced barrier disturbances in intestinal and non-intestinal epithelial systems. Evidence from intestinal injury models (Jiang et al., [198]) demonstrates that mislocalization of claudin-1 disrupts ion-selective pore formation, while occludin destabilization reduces junctional fence function, as observed in BPA-exposed epithelial cells by Pan et al. [199]. ZO-1 separation from actin filaments decreases the mechanical stress on the apical junctional complex, a finding also documented in vascular injury models by Zhuravleva et al. [200]. These studies offer indirect but supportive evidence relevant to understanding how MNPLs may influence the developing intestinal barrier.

These molecular shifts widen the paracellular cleft, increasing permeability to solutes and particle-associated contaminants. Similar patterns have been observed in Caco-2 monolayers, as described by van Breemen and Li [201], although these findings represent in vitro approximations rather than direct evidence from developing gut tissue. Developmental immaturity further modifies these processes: immature epithelia express lower levels of claudin-1, occludin and ZO-1, and these proteins undergo more dynamic regulation, consistent with the concept of developmental barrier instability described by Keaney and Campbell [193]. In vivo studies confirm that exposure to polystyrene nanoplastics reduces tight junction protein expression in a dose-dependent manner. For example, Li et al. [202] reported significant decreases in claudin-1, occludin and ZO-1 after chronic PS-NP ingestion, while He et al. [203] showed that PS-NPs intensify LPS-induced duodenal permeability via ROS-driven NF-κB/NLRP3 activation. These findings support the broader conclusion of Ma et al. [204] that MNPLs disturb tight junctions through oxidative stress, inflammation and mitochondrial dysfunction. When applied to early development, these mechanisms should be interpreted as inferred from related epithelial systems, not as direct demonstrations.

The consequences of junctional loosening are particularly pronounced during the early stages of development, when the tight junctions are assembling into their mature configuration. This vulnerability was highlighted in developmental barrier studies by Keaney and Campbell [193]. Reduced claudin expression, rapid occludin turnover, and incomplete ZO-1–actin coupling create a more permeable barrier, which is consistent with the immature epithelial phenotypes described by Grund and Grümmer [205]. Under these conditions, contaminants released from MNPLs or mobilized by natural organic matter can more readily traverse the paracellular route. In their 2025 report, Ma et al. [204] also noted paracellular leakiness in other biological barriers, including the blood–brain barrier. However, these findings are more indicative of cross-barrier analogies than direct evidence from the developing intestine. Even modest disturbances to tight junction organization may therefore increase pollutant entry during vulnerable developmental stages, as emphasized by Jonusaite and Himmerkus [206], although these conclusions remain partly inferential.

An additional layer of vulnerability arises from the dynamic turnover of the mucus layer, which is poorly synchronized in early life, as described in mucosal development studies [205]. The immaturity of mucus secretion and renewal creates transient gaps that expose the epithelium and allow microplastic–contaminant complexes to accumulate near the cell surface. This interpretation is supported by impaired mucosal clearance observed in mice exposed to PS-NPs by Li et al. [202], although these findings represent animal-model evidence. The developing microbiota also modifies mucus structure by producing enzymes and metabolites that degrade mucin, altering charge distribution and the residence time of particulate contaminants. This is supported by the findings of Zhang et al. [207] regarding the gut–liver axis. These microbiota-driven changes indirectly influence tight junction regulation via microbial metabolites such as short-chain fatty acids, which modulate epithelial signaling and barrier maturation. Such microenvironmental changes have been shown to promote local accumulation of MNPLs, increasing the likelihood of epithelial contact. Similar processes have been noted in BPA-induced mucus disruption in porcine epithelial models (IPEC-J2) by Pan et al. [199], which again provide indirect evidence rather than direct observations from the developing human gut.

Importantly, immature mucosal immune cells further contribute to this vulnerability, as dendritic cells and macrophages more frequently extend luminal sampling processes during early development, a behavior described by Grund and Grümmer [205] in mucosal immunology studies. These findings should be interpreted as supportive evidence, not as direct demonstrations of MNPL behavior in the developing intestine.

This increased antigen sampling reflects an adaptive but functionally immature immune surveillance system that prioritizes environmental sensing over barrier stability. Such enhanced sampling increases the probability that microplastic-associated contaminants will be internalized and transported into deeper tissues, consistent with animal-model findings in mice exposed to PS-NPs reported by Li et al. [202]. Concurrently, immature epithelial signaling via cytokines and chemokines is highly responsive to particulate stimuli, supported by NF-κB-mediated inflammatory activation described by He et al. [203]. These signaling disturbances weaken tight junction integrity and reinforce the permeability–inflammation feedback loop described by Ma et al. [204]. The interaction between mucus dynamics, microbiota activity and early mucosal immunity therefore creates a developmental window of heightened susceptibility to MNPL-related pollutants, as indicated by the studies of Li et al. [202], He et al. [203] and Zhang et al. [207].

At the structural level, the mucus layer during early development contains a less compact and less sulphated MUC2 network. Johansson et al. [208] demonstrated that immature MUC2 forms a looser polymeric mesh with reduced steric and electrostatic filtering capacity. Similarly, Fan et al. [209] reported that early-life mucus exhibits diminished sulphation and lower glycan density, which weakens its ability to exclude particulate matter. This results in a diffusion barrier that is more permeable, where particle mobility is primarily governed by size exclusion rather than electrostatic repulsion. This immature biochemical architecture enables MNPLs, particularly those composed of polystyrene, polyethylene and PVC, to diffuse more readily through mucus. This interpretation aligns with the enhanced penetration of polystyrene MNPLs observed by Cui et al. [210] in intestinal model systems, which provides indirect evidence relevant to early-life physiology. Li et al. [211] investigated whether increased human exposure to microplastics could be contributing to the rising incidence of early-onset colorectal cancer by disrupting the protective colonic mucus barrier, altering the way gut microbiota interact, and promoting processes involved in colorectal carcinogenesis.

In addition to the structure of the mucus, the neonatal epithelial glycocalyx also contributes to this vulnerability. As Frey et al. [212] describe, it contains fewer sialylated and fucosylated residues, which weakens the electrostatic exclusion zone that would normally repel foreign particles. This underdeveloped glycan architecture reduces the glycocalyx’s ability to prevent direct contact between luminal particles and the apical membrane. This is consistent with the impaired mucosal protection observed in early-life models by Fan et al. [209]. Reduced glycan heterogeneity may also limit the binding of protective mucins and antimicrobial peptides, thereby lowering barrier function even further. Negatively charged or amphiphilic MNPLs—such as those modified by humic substances or bile salts—can more easily adhere to or traverse the glycocalyx. This interpretation is consistent with the enhanced epithelial interaction of polystyrene MNPLs reported by Cui et al. [210] in in vitro intestinal systems. Increased adhesion raises the likelihood of epithelial uptake, as observed by Chen et al. [213] in marine medaka larvae exposed to environmentally relevant microplastic concentrations, providing supportive evidence from animal models. Many of the cited models (Caco-2, renal epithelia, BBB, burn-injury models) provide indirect insight and do not fully reflect the physiology of the developing gut.

Studies have demonstrated that the microbiota present in early life further modulate these interactions by producing glycosidases and mucin-modifying metabolites that reshape the mucus-glycocalyx interface, as described by Fan et al. [209]. Local glycan chain degradation creates transient low-density regions that act as molecular entry points. This is consistent with the observation of microbiota-driven mucus thinning in microplastic-exposed mice by Zhai et al. [214]. These microbiota-derived enzymatic activities also create spatial heterogeneity in mucus viscosity, forming “microchannels” that facilitate particle penetration. MNPLs with biofilm-like coronas—including those colonized by early gut bacteria—can accumulate within these microdomains, a behavior consistent with the microbial-plastic interactions proposed by Li et al. [211] in the context of colorectal cancer risk. This creates a permissive environment in which contaminants carried by plastics gain direct access to epithelial receptors and junctional complexes. This pathway is also supported by PS-NP-induced barrier disruption, as described by Cui et al. [210].

### 5.3. Molecular Signaling at Tight Junctions and Mucus Interfaces in the Developing Gut Barrier

As the intestinal barrier is not fully formed in developing organisms, MNPLs and their associated contaminants can exploit immature signaling pathways and weakened structural defenses. This amplifies toxicological risk. This immaturity affects both the architecture of epithelial tight junctions and the organization of the mucus layer, both of which are still undergoing dynamic remodelling during early life. Signaling pathways that regulate mucus production, such as the epidermal growth factor receptor (EGFR), interleukin-22 (IL-22) and Notch pathways, operate with reduced precision in developing organisms. This results in irregular MUC2 cross-linking and a thinner, less protective mucus layer [208,209]. Concurrently, tight junction complex maturation—involving essential proteins like claudins, occludin, and ZO-1—is incomplete, leading to increased paracellular permeability and reduced barrier selectivity.

MNPLs can further disrupt these immature pathways. For example, polystyrene particles interfere with epithelial growth factor signaling, while PVC-associated additives alter cytokine-dependent mucin expression, thereby weakening mucus defenses in early life [215]. Additionally, these particles may indirectly affect tight junction integrity by influencing oxidative stress and inflammatory signaling cascades, which regulate junctional protein expression and assembly. These disruptions create transient low-density regions that allow particle-contaminant complexes to approach the epithelium more easily. The concurrent weakening of mucus viscosity and tight junction cohesion acts synergistically to further enhance epithelial exposure to environmental particulates.

As summarized in Table 3, the interplay between plastic physicochemistry, biocorona formation, mucus rheology, and epithelial biomechanics gives rise to a uniquely permissive environment for pollutant uptake during early development. These processes emphasize the heightened vulnerability of the developing gut barrier, where even low-level exposure can have a disproportionately high biological impact due to incomplete maturation of the barrier and signaling plasticity.

Studies have demonstrated that the regulation of tight junctions is fragile during development, as the assembly of claudin–occludin–ZO-1 complexes via MLCK-, RhoA/ROCK- and PKC-dependent pathways is less stable during early life [212]. This incomplete maturation of junctional signaling results in reduced epithelial cohesion and impaired barrier selectivity in the intestines of neonates and juveniles. Furthermore, MNPLs -derived chemicals such as bisphenols and phthalates activate oxidative and inflammatory pathways that modify junctional phosphorylation and weaken cytoskeletal anchoring, thereby increasing paracellular permeability [199,202]. These signaling disturbances are often linked to ROS overproduction, NF-κB activation, and altered kinase activity, all of which contribute to tight junction destabilization. These effects are exacerbated when MNPLs act as carriers for adsorbed metals or hydrophobic pollutants, further destabilizing junctional signaling and increasing oxidative stress [199,216].

Consequently, the immature intestinal barrier becomes increasingly vulnerable to the penetration of particle-associated toxicants during critical developmental windows.

Further studies have demonstrated that crosstalk between mucus-derived cues and epithelial signaling amplifies this developmental vulnerability. Mucin fragments, microbial metabolites and luminal cytokines modulate tight junction (TJ) expression, while epithelial stress responses simultaneously alter mucin secretion [209]. This communication between the mucus layer and the epithelial junctional machinery is vital for maintaining barrier homeostasis under physiological conditions; however, it is particularly unstable during early development. MNPLs exacerbate this pathological process by inducing ROS- and cytokine-driven signaling in gastric and hepatointestinal tissues, thereby weakening mucus and junctional defenses simultaneously [207,216]. In parallel, inflammatory mediators may disrupt goblet cell function and alter MUC2 organization, further deteriorating mucus barrier integrity. This bidirectional interaction creates transient windows of opportunity during which particle–contaminant complexes can exploit weaknesses in both protective layers (Table 3).

The biophysical properties of neonatal mucus, including lower viscosity, reduced elasticity, and diminished mesh density, further enhance particle mobility. This is consistent with the loose MUC2 architecture observed in early life [208]. This immaturity in the mucus layer’s rheological properties limits its ability to efficiently trap and immobilize foreign particles. Smooth-surfaced MNPLs move with minimal drag, while biofilm-coated particles remain mobile due to sparse mucin entanglement [199]. Reduced ionic strength and altered luminal pH in the immature gut also facilitate the movement of negatively charged or hydrophobic plastics [211]. These physicochemical conditions favor the deeper penetration of MNPLs towards the epithelial surface.

At the epithelial interface, immature enterocytes exhibit lower cortical tension and reduced cytoskeletal stiffness, as well as incomplete junctional sealing. This facilitates both paracellular and transcellular particle translocation [212]. The underdeveloped glycocalyx and reduced membrane rigidity that are characteristic of early life also decrease the mechanical resistance to particle adhesion and uptake. Plastic-associated pollutants exacerbate this increased permeability by inducing oxidative stress and altering membrane lipid composition, thereby further softening epithelial mechanics [216]. These alterations may also impair membrane trafficking and intracellular signaling pathways involved in epithelial repair and barrier recovery. These developmental characteristics make early life a period of heightened susceptibility to MNPLs exposure. The immature mucus layer, simplified glycocalyx and unstable tight-junction signaling provide multiple entry points for particle-associated contaminants [199,208,209]. The current body of evidence suggests that the developing gastrointestinal barrier is a uniquely permissive biological interface, where structural immaturity and environmentally induced signaling disturbances converge to increase toxicological risk.

### 5.4. Early-Life Susceptibility Is Due to Underdeveloped Detoxification Systems and Epithelial Turnover

The immaturity of detoxification systems and the slow epithelial turnover characteristic of the neonatal gut greatly increase susceptibility in early life, a pattern consistent with evidence showing that early exposures can transiently prime or suppress metabolic pathways [218]. During this developmental period, intestinal defense mechanisms are still maturing, making the gut particularly vulnerable to persistent environmental stressors, including MNPLs. Phase I and phase II metabolic pathways, particularly cytochrome P_450_ enzymes, glutathione-dependent conjugation systems, and UDP-glucuronosyltransferases, operate at significantly reduced capacity during early development. This is reflected in the low baseline expression of CYP1, CYP2 and CYP3 family members in developing human tissues [219]. Consequently, enterocytes have limited capacity to neutralize contaminants associated with plastics, such as bisphenols, phthalates, brominated flame retardants and adsorbed polycyclic aromatic hydrocarbons. All of these require CYP-dependent activation or detoxification [220,221]. At the same time, the expression of xenobiotic efflux transporters, including ATP-binding cassette (ABC) family proteins, is lower in early life. This reduces the capacity to eliminate toxicants carried by MNPLs [222,223]. The slow turnover of the neonatal epithelium prolongs exposure further, as damaged or stressed enterocytes remain in place for longer, increasing the likelihood of particle-induced oxidative stress, membrane disruption, and junctional destabilization [218]. MNPLs with reactive or pollutant-rich coronas exacerbate these vulnerabilities by delivering concentrated chemical loads directly to cells lacking the necessary metabolic and transport machinery for efficient detoxification [224]. These developmental limitations significantly increase the intracellular retention and biological activity of toxicants associated with plastics.

Although cytochrome P_450_ enzymes are the main machinery involved in xenobiotic metabolism, they are expressed and catalysed at markedly lower levels during early life [219]. Key isoforms involved in the metabolism of chemicals associated with plastics, including CYP1A1, CYP2C, CYP2E1 and CYP3A, are expressed at only a fraction of adult levels. Furthermore, developmental differences in membrane composition, cofactor availability, and enzyme maturation may compromise catalytic performance in neonatal tissues. The structural diversity and membrane-anchored configuration of CYP enzymes also influence their catalytic efficiency [225,226,227]. This immaturity restricts the hydroxylation and detoxification of compounds leaching from plastics, including bisphenol A, styrene monomers, phthalates, and brominated flame retardants [220]. Furthermore, MNPLs can suppress CYP expression by inducing oxidative stress and inflammatory signaling, thereby creating a feedback loop in which reduced metabolic capacity increases the intracellular burden of pollutants, which in turn further inhibits CYP activity [218]. Inflammatory mediators such as NF-κB and cytokine-associated signaling pathways may also repress CYP gene transcription, contributing to prolonged xenobiotic persistence within epithelial cells.

Glutathione S-transferases (GSTs) are another important class of detoxification enzymes. They catalyse the conjugation of electrophilic contaminants with glutathione (GSH), facilitating the neutralization and excretion of these contaminants [221]. In neonates, however, GST expression and intracellular GSH levels are substantially lower, reducing the capacity to detoxify reactive, plastic-associated chemicals, such as aldehydes, quinones, and oxidized additives [220]. MNPLs exacerbate this limitation by generating reactive oxygen species that rapidly deplete GSH reserves. MNPLs with metal-rich coronas (e.g., iron, copper and zinc) may also catalyse Fenton-like reactions, thereby overwhelming the already limited GST-GSH detoxification axis [224]. As oxidative stress accumulates, impaired redox buffering further destabilizes epithelial membranes, mitochondrial function, and tight-junction integrity. This links detoxification failure directly with barrier dysfunction.

Studies have shown that ABC transporters, including ABCB1 (P-gp), ABCC2 (MRP2) and ABCG2 (BCRP), play a central role in exporting xenobiotics and their metabolites from enterocytes [222]. However, in early life, these transporters are expressed at lower levels and exhibit reduced ATP-dependent efflux capacity [223,228]. Consequently, contaminants carried by MNPLs accumulate intracellularly rather than being efficiently removed. Furthermore, MNPLs can interfere with ABC transporter function by altering membrane fluidity or competitively inhibiting transporter binding sites [229]. However, some studies suggest that polystyrene particles do not universally inhibit transporter activity [230]. Hydrophobic polymers, such as polyethylene and polypropylene, can integrate into the apical membrane when coated with bile acids or dietary lipids, thereby impairing transporter localization and function [222]. This disruption to membrane-associated transport processes may further enhance the retention of toxicants inside cells and prolong epithelial exposure to plastic-derived chemicals.

In addition to impairing metabolic and efflux pathways, MNPLs interfere directly with the cellular machinery responsible for maintaining epithelial renewal and detoxification homeostasis. Many polymers, particularly polystyrene, PVC and polyurethane, release additives and monomers that can disrupt mitochondrial function. This reduces the availability of ATP, which is required for CYP catalysis, GST conjugation and ABC transporter activity [220,221]. Mitochondrial dysfunction may also increase ROS generation and impair cellular energy-sensing pathways, further compromising epithelial resilience. Nanoplastics can accumulate within stem cell niches located at the base of intestinal crypts. There, their oxidative and electrophilic properties may impair Wnt- and Notch-dependent proliferation, slowing epithelial regeneration further still [231]. Particles carrying bioactive coronas enriched in microbial enzymes, bile acids or dietary lipids can penetrate deeper into crypt regions, altering the redox balance and the epigenetic regulators that control the expression of detoxification genes [224]. These combined effects create a vulnerability that is compounded by the fact that plastics deliver concentrated chemical stressors to cells with limited metabolic capacity while simultaneously weakening the regenerative and energetic systems required for recovery following exposure.

**Figure 4:**
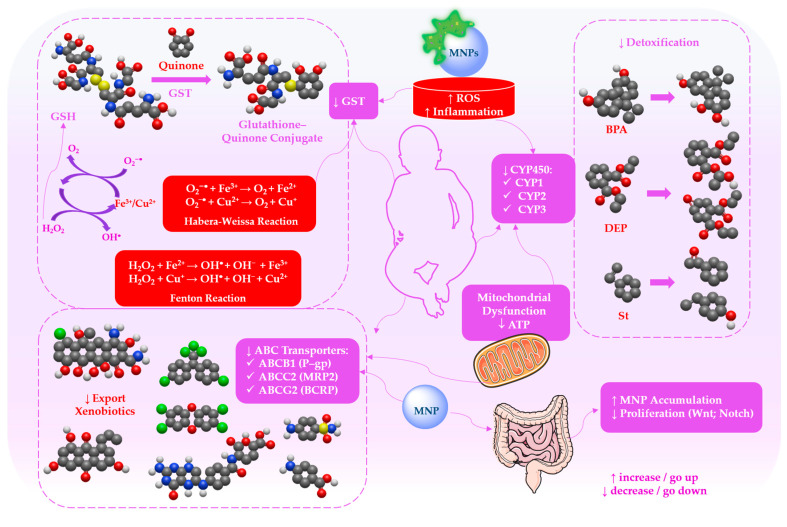
Immaturity of detoxification systems and epithelial turnover in neonates. The neonatal intestine is particularly vulnerable to micro- and nanoplastics (MNPs) due to immature detoxification systems, reduced xenobiotic efflux, and slow epithelial turnover. Limited activity of CYP enzymes, GST/GSH pathways, and ABC transporters promotes the accumulation of plastic-associated contaminants, while MNP-induced oxidative stress and mitochondrial dysfunction further impair cellular defense and barrier integrity. Image provided by Servier Medical Art (https://smart.servier.com/), licensed under CC BY 4.0 (https://creativecommons.org/licenses/by/4.0/, accessed on 15 April 2026). Abbreviations: ABC—ATP-binding cassette (transporter family involved in xenobiotic efflux); ABCB1 (P-gp)—ATP-binding cassette subfamily B member 1 (P-glycoprotein); ABCC2 (MRP-2)—ATP-binding cassette subfamily C member 2 (multidrug resistance-associated protein 2); ABCG2 (BCRP)—ATP-binding cassette subfamily G member 2 (breast cancer resistance protein); ATP—adenosine triphosphate; BPA—bisphenol A; CYP1—cytochrome P450 family 1; CYP2—cytochrome P450 family 2; CYP3—cytochrome P450 family 3; CYP450—cytochrome P450 (enzyme superfamily involved in Phase I metabolism); DEP—diethyl phthalate; GSH—glutathione; GST—glutathione S-transferase; MNPs—micro- and nanoplastics; Notch—notch signaling pathway; St—styrene; Wnt–wingless/integrated signaling pathway.

Figure 4 illustrates how immature neonatal detoxification systems and reduced epithelial turnover increase susceptibility to MNPLs exposure. It emphasizes that limited xenobiotic metabolism, impaired efflux capacity and weakened barrier renewal promote the intracellular accumulation of contaminants and enhance oxidative stress-related cellular damage in early life.

Furthermore, the gut barrier exhibits reduced structural integrity, limited detoxification capacity and slower epithelial renewal in early life [212]. These developmental characteristics create a highly permissive environment for the penetration and persistence of MNPLs within intestinal tissues. The loose mucin network, simplified glycocalyx and unstable junctional architecture allow diverse plastic particles, particularly those carrying bioactive coronas, to reach epithelial cells with minimal resistance [208,209]. Once in contact with epithelial surfaces, immature metabolic systems and weak efflux machinery are unable to efficiently neutralize or remove MNPLs -associated chemicals, enabling their accumulation and promoting prolonged cellular stress [218]. Furthermore, MNPLs intensify this vulnerability by disrupting mitochondrial energy production and impairing stem cell-driven epithelial regeneration [231]. Thus, the available evidence indicates that the developing intestine is a particularly sensitive toxicological target, where incomplete barrier maturation, impaired detoxification pathways and reduced regenerative capacity combine to increase the biological effects of plastic exposure in the earliest stages of life.

## 6. Oxidative Stress Pathways Activated by Combined Exposure

### 6.1. Generation of Reactive Oxygen Species (ROS) by Metals, Pesticides and Perfluoroalkyl Substances (PFAS) Carried by MNPLs

When metals are transported on MNPLs surfaces and enter the cellular environment, they can initiate intense oxidative stress through classical Fenton and iron-catalysed Haber-Weiss reactions. Transition metals such as Fe^2+^ and Cu^+^ catalyse the conversion of basal hydrogen peroxide into highly reactive hydroxyl radicals. This mechanism was first described by Kehrer [232] and subsequently clarified in analyses of the Haber-Weiss cycle by Koppenol [233] and Liochev and Fridovich [234]. As metal-loaded MNPLs accumulate within endosomes and mitochondria, they destabilize complexes I and III of the electron transport chain (ETC), thereby promoting electron leakage and increasing superoxide formation.

In parallel, released metal ions may bind directly to mitochondrial proteins and phospholipids, further impairing oxidative phosphorylation and mitochondrial membrane stability. This initial burst of oxidation activates Nrf2-Keap1 signaling as a compensatory antioxidant response. However, persistent metal exposure overwhelms endogenous defense systems and shifts redox signaling towards pro-oxidant pathways involving p38 and JNK MAPKs. This is consistent with the broader oxidative stress framework described by Kovacs et al. [7]. Consequently, the intracellular environment becomes progressively more permissive to secondary ROS-generating contaminants associated with microplastic particles.

This metal-driven oxidative environment markedly increases the susceptibility of cells to pesticides that are transported by MNPLs. MNPLs act as vectors, delivering pesticides directly to epithelial membranes and endosomal compartments and bypassing normal detoxification gradients—a phenomenon highlighted in the literature on emerging contaminants by Santhanam et al. [3]. Many pesticides are metabolically activated by CYP1A1, CYP2B6 and CYP3A isoforms, generating unstable intermediates that can leak electrons, producing superoxide and hydrogen peroxide [225,235,236]. Concurrently, the activation of PKC and RhoA induced by pesticides stimulates NOX1/NOX2 complexes, thereby increasing ROS production further [237,238,239]. These NOX-dependent pathways also increase inflammatory signaling and lipid peroxidation, thereby intensifying epithelial oxidative injury. The synergy between metal-initiated ROS formation and pesticide-driven redox cycling creates a highly localized oxidative microenvironment that magnifies toxicity even at relatively low pesticide concentrations. Thus, oxidative damage may extend beyond mitochondria to affect membranes, proteins, and DNA, ultimately compromising epithelial barrier integrity and cellular viability.

The oxidative stress initiated by metals and intensified by pesticide metabolism further amplifies the ROS-generating effects of PFAS carried by MNPLs. PFAS disrupt mitochondrial inner-membrane lipids, impairing ETC complexes I and II and increasing electron leakage, as demonstrated in algal and plant models by Zhao et al. [17] and Sun et al. [18]. PFAS also activate peroxisome proliferator-activated receptor alpha (PPARα), thereby enhancing peroxisomal β-oxidation—a process that generates hydrogen peroxide—while simultaneously interfering with the nuclear factor erythroid 2-related factor 2 (Nrf2), aryl hydrocarbon receptor (AhR) and NF-κB pathways that normally coordinate antioxidant and inflammatory responses.

Subcellular analyses by Amstutz et al. [20] demonstrated that PFAS reduce catalase activity and induce ROS through mitochondrial and non-mitochondrial sources, with headgroup-dependent differences in toxicity. MNPLs coated with PFAS may also integrate into epithelial membranes, stimulating NOX4 activity and destabilizing redox-sensitive signaling pathways. This mechanism is consistent with PFAS-microplastic interactions as described by Parashar et al. [19] and with mixture-toxicity studies as reported by Sands et al. [21]. Furthermore, PFAS-induced membrane perturbations may increase cellular permeability and facilitate the intracellular accumulation of co-transported contaminants. Thus, PFAS exploit the oxidative landscape shaped by metals and pesticides, driving ROS production beyond the detoxification capacity of the developing gut.

In addition to the chemical contaminants they carry, MNPLs act as potent amplifiers of oxidative stress by physically disturbing cellular structures and redox-sensitive pathways. This phenomenon is becoming increasingly recognized in various tissues and experimental model systems [197,240]. Their rigid surfaces and nanoscale edges enable them to penetrate epithelial membranes, disrupting lipid organization and triggering NOX2- and NOX4-dependent activation through mechanosensitive signaling pathways. This is consistent with evidence showing that polyethylene MNPLs activate the TLR4/NOX2 axis and induce ROS-dependent cellular senescence [241]. Once internalized, MNPLs accumulate within lysosomes, where their poor degradability can lead to lysosomal membrane permeabilization (LMP). This process releases cathepsins that activate NLRP3 inflammasome signaling and secondary ROS generation.

These findings are consistent with studies showing that MNPLs robustly stimulate NLRP3-mediated immunotoxicity [242] and induce lysosomal dysfunction leading to necroptosis [243]. These particles also interfere with mitochondrial dynamics, altering the balance between OPA1- and DRP1-dependent fusion and fission and thereby increasing ETC electron leakage. This mechanism is supported by studies reporting DRP1-dependent mitochondrial injury in MNPLs -exposed hepatocytes [244]. At the transcriptional level, MNPLs modulate the signaling of Nrf2, HIF-1α and NF-κB, shifting cells towards a pro-oxidant phenotype even before metals, pesticides or PFAS can exert their toxic effects. This is consistent with reports of NF-κB/NLRP3 activation within the gut–liver axis following MNPLs exposure [245]. Furthermore, the sustained activation of inflammasome-associated pathways can perpetuate chronic low-grade inflammation and ROS production over extended periods. In this manner, the particles themselves create a redox-primed intracellular environment that amplifies the ROS-generating potential of the contaminants they carry [246].

These mechanisms demonstrate that MNPLs function as both carriers and catalytic amplifiers of oxidative stress. This creates a biochemical environment in which metals, pesticides and PFAS generate ROS far more efficiently than they would individually [197]. Metals initiate redox-active reactions, pesticides enhance ROS production through CYP-dependent metabolism and NOX activation, and PFAS sustain oxidative pressure by disrupting mitochondrial and peroxisomal pathways. The particles themselves also destabilize membranes, lysosomes and mitochondrial dynamics, thereby preconditioning cells for enhanced oxidative injury [247,248]. The combined effect is a multilayered, self-reinforcing ROS cascade that exceeds the antioxidant capacity of the developing gut and magnifies the toxic potential of all contaminants associated with MNPLs. This convergence of oxidative, inflammatory and mitochondrial stress pathways likely plays a central role in epithelial dysfunction, impaired barrier homeostasis, and long-term intestinal vulnerability during early developmental stages. These conclusions are supported by extensive evidence of oxidative injury, organelle dysfunction, and chronic inflammatory activation in MNPLs -exposed biological systems [243,244,245].

### 6.2. Activation of the Nrf2–Keap1 Antioxidant Response Pathway

Intense oxidative stress, generated by metals, pesticides, PFAS and MNPLs, rapidly activates the Nrf2-Keap1 antioxidant response pathway. Sun et al. [216] provided strong support for this mechanism by demonstrating that polystyrene micro- and nanoplastics markedly increase ROS levels, disrupt mitochondrial homeostasis, and activate the p62/Keap1/Nrf2 axis in gastric tissue. Under physiological conditions, Keap1 binds to Nrf2 in the cytosol and targets it for ubiquitin-mediated degradation, thereby maintaining low basal antioxidant activity. However, ROS and electrophilic contaminants can modify critical cysteine residues within Keap1, weakening its interaction with Nrf2 and enabling Nrf2 nuclear translocation. Once Nrf2 is translocated into the nucleus, it induces the expression of ARE-dependent genes such as HO-1, NQO1 and GCLC. This early adaptive response is essential for restoring redox balance and limiting oxidative injury following acute exposure to contaminants associated with plastics. The protective role of this pathway is consistent with the findings of Guo et al. [249], who demonstrated that ROS-activated Nrf2 mitigates mitochondrial damage and inflammation in hepatocytes exposed to nanoplastics, whereas Nrf2 knockdown markedly exacerbates toxicity.

However, the same MNPLs that initially activate this defense pathway can also undermine its long-term effectiveness. Wen et al. [250] reported that chronic MNPLs exposure suppresses Nrf2 signaling in the liver, thereby reducing the expression of downstream antioxidant enzymes and promoting oxidative stress. They demonstrated that prolonged ROS overload can shift Nrf2 signaling from adaptive activation to functional insufficiency, particularly when mitochondrial dysfunction and NLRP3 inflammasome activation occur simultaneously. This aligns with the findings of Sun et al. [216], who showed that persistent ROS generation disrupts mitochondrial membrane potential, ATP production, and mitochondrial dynamics, overwhelming the antioxidant system. Continuous Nrf2 activation progressively depletes glutathione reserves and alters redox-sensitive signaling pathways. Meanwhile, PFAS- and pesticide-induced lipid perturbations may destabilize the Keap1-Cul3 complex, locking cells into a chronic stress-response state. Under these conditions, antioxidant signaling becomes maladaptive, contributing to sustained oxidative imbalance rather than effective cytoprotection.

In the developing gut, where antioxidant reserves and detoxification systems are inherently limited, this maladaptive Nrf2 activation creates additional vulnerability rather than providing an effective defense. Immature enterocytes have lower baseline expression of antioxidant enzymes and reduced glutathione buffering capacity, which makes them particularly susceptible to prolonged exposure to ROS. This developmental susceptibility is consistent with the broader pattern observed within the gut-liver axis. For instance, Chen et al. [251] demonstrated that MNPLs compromise intestinal tight junctions, thereby increasing epithelial permeability and elevating circulating endotoxin levels. This subsequently activates hepatic TLR4/NF-κB/NLRP3 signaling pathways. Similarly, Zhou et al. [252] showed that MNPLs suppress SIRT1-AMPK signaling and activate TLR4/NF-κB pathways in the liver, thereby exacerbating oxidative stress and inflammation. These findings suggest that local intestinal oxidative injury can lead to systemic inflammation and metabolic disturbances that extend beyond the gastrointestinal tract.

Analysis of these processes indicates that, while essential, Nrf2 activation in the early-life intestine is insufficient. Although the pathway initially provides protection against ROS accumulation, the sustained oxidative burden imposed by MNPLs and their associated contaminants quickly exceeds antioxidant capacity. Wen et al. [250] demonstrated that, when Nrf2 signaling is repressed or exhausted, activation of the NLRP3 inflammasome intensifies, resulting in increased levels of IL-1β and caspase-1. This creates a redox-imbalanced environment in which antioxidant defenses are activated but ultimately depleted, thereby increasing the immature gut’s susceptibility to MNPL-driven toxicity.

The involvement of the gut-liver axis, as demonstrated by Chen et al. [251] and Zhou et al. [252], further exacerbates this vulnerability by propagating systemic oxidative and inflammatory signaling. Consequently, oxidative stress evolves from a transient adaptive response into a chronic pathogenic process associated with epithelial dysfunction and inflammatory injury.

**Figure 5:**
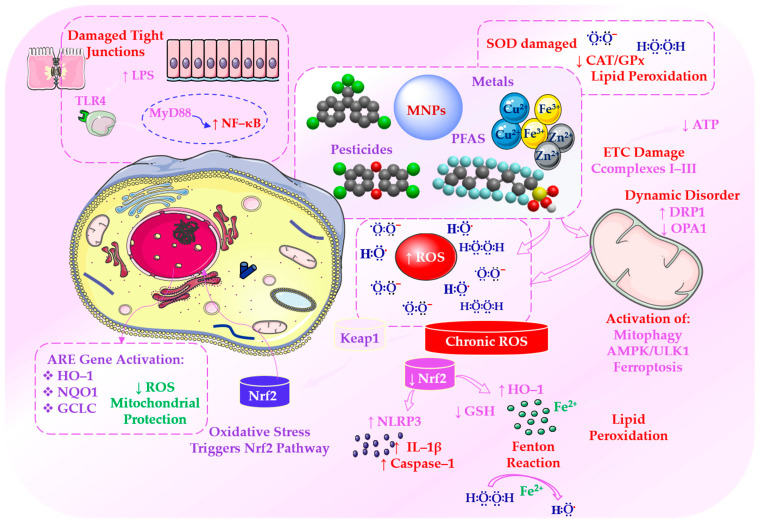
Oxidative stress-driven disruption of Nrf2 signaling and mitochondrial dysfunction induced by micro- and nanoplastics. Micro- and nanoplastics (MNPs) and associated pollutants induce excessive ROS production, activating the Nrf2–Keap1 antioxidant pathway. Although Nrf2 initially promotes protective responses, chronic oxidative stress leads to pathway exhaustion, glutathione depletion, and impaired antioxidant defense. Mitochondrial dysfunction further enhances ROS generation, while inflammatory signaling amplifies oxidative damage, contributing to cellular dysfunction and increased vulnerability of the developing intestine. Image provided by Servier Medical Art (https://smart.servier.com/), licensed under CC BY 4.0 (https://creativecommons.org/licenses/by/4.0/, accessed on 15 April 2026). Abbreviations: AMPK—AMP-activated protein kinase; ARE—antioxidant response element; ATP—adenosine triphosphate; CAT—catalase; DRP1—dynamin-related protein 1; ETC—electron transport chain; GCLC—glutamate–cysteine ligase catalytic subunit; GPx—glutathione peroxidase; GSH—glutathione; HO-1—heme oxygenase 1; IL-1β—interleukin 1 beta; Keap1—kelch-like ECH-associated protein 1; LPS—lipopolysaccharide; MyD88—myeloid differentiation primary response protein 88; MNPs—micro- and nanoplastics; NF-κB—nuclear factor kappa B; NLRP3—NOD-like receptor family, pyrin domain containing 3; NQO1—NAD(P)H quinone dehydrogenase 1; Nrf2—nuclear factor erythroid 2–related factor 2; OPA1—optic atrophy 1; PFAS—per- and polyfluoroalkyl substances; ROS—reactive oxygen species; SOD—superoxide dismutase; TLR4—toll-like receptor 4; ULK1—unc-51 like autophagy activating kinase 1.

Figure 5 illustrates the mechanisms by which MNPLs induce oxidative stress through the generation of excessive ROS, the disruption of Nrf2-mediated antioxidant defenses and mitochondrial dysfunction. It also shows how oxidative stress interacts with inflammatory signaling pathways, resulting in impaired cellular homeostasis and an increased susceptibility of the developing intestine to toxic injury.

The presence of MNPLs disrupts endogenous antioxidant systems by interacting directly with cellular proteins and enzymes. These particles are now recognized as widespread environmental contaminants that can enter cells and interact directly with protein structures [253,254,255]. MNPLs can profoundly alter the structure and function of key antioxidant enzymes, particularly superoxide dismutase (SOD). Smaller particles induce loosening of the protein backbone, whereas larger particles tighten peptide-chain organization, demonstrating clear size-dependent toxicity [254]. These structural alterations reshape the intracellular redox landscape by compromising endogenous antioxidant defenses and increasing lipid peroxidation, while simultaneously activating inflammatory and ferroptotic pathways, including JNK/HO-1 signaling [256,257]. This further reduces the ability of cells to neutralize ROS generated by plastic-associated contaminants.

The initial ROS burst, generated by MNPLs and adsorbed environmental contaminants (including metals, pesticides, and PFAS), activates redox-sensitive transcription factors and contributes to oxidative stress-mediated cellular injury [253,258]. This oxidative burden is consistent with evidence showing that modulation of SOD activity, whether through MNPLs interference or therapeutic nanoparticle-mediated delivery, directly influences cellular resilience under oxidative stress [259]. The continuous environmental degradation of plastics into MNPLs, combined with atmospheric ageing processes, enhances their biological reactivity and toxic potential in multiple tissues, including the nervous system, reproductive organs, vascular endothelium, and the gastrointestinal tract [258,260,261,262].

This early compensatory response is consistent with the oxidative stress framework proposed by Hu and Palić [263], who identified ROS overproduction and MAPK activation as central events in the early stages of MNPLs toxicity. However, sustained exposure disrupts this balance, with excessive superoxide overwhelming SOD activity and leading to the accumulation of hydrogen peroxide that exceeds the detoxification capacity of catalase (CAT) and glutathione peroxidase (GPx). Kadac-Czapska et al. [264] similarly highlighted this pattern in their review of microplastic-induced oxidative stress. As antioxidant buffering capacity decreases, oxidative damage progressively extends to mitochondrial membranes, proteins, and nucleic acids.

Chronic Nrf2 activation also stimulates the continuous expression of HO-1, which increases the levels of intracellular free iron through haem degradation. This process fuels Fenton chemistry and amplifies hydroxyl radical formation, shifting the antioxidant system from a coordinated defense mechanism to one of enzymatic imbalance and pro-oxidant signaling. Jia et al. [265] observed similar maladaptive Nrf2 responses, demonstrating that MNPLs disrupt Keap1/Nrf2/ARE signaling and impair the regulation of antioxidant genes under prolonged oxidative stress. Consequently, the prolonged activation of antioxidant pathways paradoxically contributes to the amplification of ROS and cellular injury.

The collapse of antioxidant defenses is closely associated with mitochondrial dysfunction, a key hallmark of microplastic-induced toxicity. MNPLs accumulate within mitochondria, disrupting inner membrane integrity and impairing ETC complexes I-III [266,267]. This promotes electron leakage and uncontrolled superoxide generation. These mechanisms align with the findings of Yang et al. [268], who demonstrated that oxidative stress-driven mitochondrial damage triggers ferroptosis and ferritinophagy in lung tissue exposed to polystyrene nanoparticles. Furthermore, Milillo et al. [269] reported that MNPLs induce mitochondrial depolarization, senescence, and apoptosis in human alveolar epithelial cells. This provides additional evidence linking mitochondrial injury with oxidative collapse. These mitochondrial abnormalities also compromise ATP synthesis, limiting the energy available for cellular repair and detoxification processes.

MNPLs -associated contaminants also destabilize cardiolipin, which impairs respiratory supercomplexes and ATP synthesis. Altered mitochondrial dynamics, mediated by dysregulated DRP1-dependent fission and OPA1-regulated fusion, exacerbate ROS production further and trigger mitophagy. When mitochondrial damage becomes excessive, cytochrome c release can trigger mitochondrial apoptosis. This mechanism aligns with the findings of Huang et al. [270], who demonstrated that polystyrene MNPLs activate AMPK/ULK1-dependent mitophagy in neuronal cells. As ETC efficiency declines, cells shift towards anaerobic metabolism. This reduces the availability of NADPH required for GPx and glutathione reductase activity, ultimately accelerating antioxidant failure. Real-time imaging studies by Zhao et al. [271] further confirmed that MNPLs induce rapid, mechanically amplified ROS bursts in alveolar cells that overwhelm antioxidant defenses. The resulting energetic collapse intensifies the redox imbalance further and promotes irreversible cellular injury.

In addition to causing oxidative stress and mitochondrial dysfunction, MNPLs disrupt broader cellular homeostasis, affecting processes such as neurotoxicity, immune dysregulation, and cross-organ communication. Several studies demonstrate that MNPLs can penetrate the central nervous system and induce neuronal injury via oxidative stress, MAPK activation, and metal-dependent cell death pathways. For instance, Chen et al. [272] revealed that polystyrene MNPLs activate ERK/MAPK-mediated cuproptosis, which is characterized by copper accumulation, DLAT oligomerization, and mitochondrial collapse. This ultimately impairs synaptic plasticity and cognitive function. Complementary findings by Dai et al. [273] suggest that exposure to environmental oxidants, such as ozone, intensifies MNPLs -induced neuronal pyroptosis. This highlights the nervous system’s vulnerability to combined oxidative and inflammatory insults. These observations are consistent with the adverse outcome pathways proposed by Hu and Palić [274], who emphasized that MNPLs activate highly conserved oxidative and inflammatory cascades across tissues. This indicates that systemic propagation of oxidative signaling may have consequences extending far beyond the gastrointestinal tract.

MNPLs interfere with cellular quality-control systems, such as autophagy, mitophagy and ferroptosis, which increase tissue injury. Huang et al. [270] demonstrated that polystyrene MNPLs activate AMPK/ULK1-dependent mitophagy in neuronal and dopaminergic cells, representing an attempt to compensate for and remove damaged mitochondria. However, when mitochondrial injury becomes excessive, MNPLs promote transitions towards regulated necrotic pathways. In lung tissue, Yang et al. [268] showed that oxidative stress-driven mitochondrial damage induces ferritinophagy and ferroptosis, thereby linking iron dysregulation directly to lipid peroxidation. Milillo et al. [269] reported similar patterns of oxidative imbalance and apoptosis in human alveolar epithelial cells. These findings suggest that MNPLs not only disrupt antioxidant defenses, but also cellular systems responsible for maintaining mitochondrial integrity and iron homeostasis. This contributes to multi-organ toxicity. Convergence of oxidative stress, mitochondrial dysfunction, and impaired cellular quality control, therefore, represents a central mechanism underlying the systemic toxicity of MNPLs.

These mechanisms demonstrate how MNPLs can weaken enzymatic antioxidant defenses and compromise mitochondrial function simultaneously, thereby creating a cycle of self-reinforcing ROS overproduction, metabolic collapse and redox imbalance. This cycle is particularly damaging during early development when antioxidant reserves, detoxification pathways and mitochondrial resilience are not fully mature. Consequently, the developing gut becomes highly susceptible to persistent oxidative injury, inflammatory signaling and epithelial dysfunction following exposure to MNPLs and their associated contaminants.

## 7. Inflammatory Signaling Triggered by Combined Microplastic and Contaminant Exposure

MNPLs act as dual inflammatory stimuli, combining the physical disruption of the epithelium with the chemical activation of innate immune receptors. This mechanism aligns with Li and Wu’s [274] fundamental description of pattern-recognition receptors (PRRs), which emphasizes that PRRs detect pathogen-associated molecular patterns (PA MNPLs) and damage-associated molecular patterns (DA MNPLs), thereby initiating downstream immune signaling cascades. Their classification of PRRs into Toll-like receptors (TLRs), NOD-like receptors (NLRs), RIG-I-like receptors (RLRs), C-type lectin receptors (CLRs), and AIM2-like receptors (ALRs) provides a structural framework for understanding how MNPLs, which induce epithelial microinjury and carry adsorbed contaminants, are recognized as danger-associated stimuli. This conceptual model is consistent with the broader immunological overviews presented by Riera Romo et al. [275] and Kaur and Secord [276], who emphasized the pivotal role of PRRs in linking innate and adaptive immunity during tissue injury. The activation of TLRs 2, 4 and 9 by MNPLs is also consistent with the canonical TLR signaling mechanisms described by Takeda and Akira [277], who demonstrated that TLRs recruit adaptor proteins such as MyD88 and TRIF to initiate inflammatory signaling.

Taguchi and Mukai [278] further complemented these findings by demonstrating that TLR signaling is tightly coupled to membrane trafficking dynamics. This helps to explain why persistent microplastic exposure can maintain chronic receptor activation by delivering ligands continuously to endosomal compartments. Consequently, the combination of epithelial barrier disruption and persistent PRR stimulation creates a highly sensitized inflammatory environment in the developing gut. This interaction is similar to that observed by Thaiss et al. [279], who demonstrated that epithelial barrier damage increases the sensitivity of PRRs to microbial products—a mechanism that is highly relevant to exposure to microplastic-associated endotoxins and contaminants.

Once TLRs are activated, the resulting intracellular signaling primarily converges on the NF-κB and MAPK pathways. This is consistent with the work of Troutman et al. [280], who demonstrated that TLR activation induces NF-κB-dependent transcription of pro-inflammatory genes, while phosphoinositide 3-kinase (PI3K) acts as a regulatory mechanism that limits excessive inflammation. Ni et al. [281] further supported this regulatory mechanism by showing that BCAP-mediated PI3K activation suppresses excessive TLR signaling. These findings suggest that chronic microplastic exposure may overwhelm physiological anti-inflammatory feedback systems, thereby promoting sustained inflammatory activation. Several toxicological studies provide direct experimental evidence for microplastic-induced activation of the MAPK and NF-κB pathways. For instance, Chen et al. [282] demonstrated that polystyrene MNPLs activate MAPK/NF-κB signaling in macrophages in a surface chemistry-dependent manner.

Similarly, Tang et al. [283] showed that MNPLs exacerbate lipopolysaccharide (LPS)-induced necroptosis and inflammation through ROS/MAPK signaling in the spleens of mice. Cui et al. [284] further reported that MNPLs activate TLR2-dependent inflammation in the hepatopancreas of carp through excessive ROS accumulation. Conversely, Antunes et al. [285] demonstrated TLR4/p38-mediated inflammatory activation in human intestinal epithelial cells and mouse microglia. These studies suggest that MNPLs activate receptor-level (TLR2/TLR4) and intracellular (MAPK/NF-κB) inflammatory pathways via the activation of macrophages, the generation of ROS and the formation of synergistic interactions with endotoxins. This coordinated inflammatory response substantially amplifies tissue susceptibility to oxidative and epithelial injury.

Persistent activation of NF-κB and MAPKs leads to pronounced upregulation of IL-1β, IL-6 and TNF-α, which is consistent with the inflammasome-priming mechanisms described by Li and Wu [274]. MNPLs also trigger NLRP3 inflammasome activation via lysosomal rupture, ROS generation and ionic imbalance—mechanisms that are also emphasized in the wider PRR literature by Riera Romo et al. [275]. These cytokines reinforce oxidative stress through NOX-dependent ROS production, the inhibition of antioxidant enzymes, and mitochondrial dysfunction. This establishes a self-sustaining oxidative-inflammatory loop. Notably, ROS generated downstream of cytokine signaling further sensitize PRRs and inflammasome pathways, creating a potent amplification mechanism. In immature tissues, where antioxidant capacity and epithelial repair mechanisms are underdeveloped, this loop can be particularly damaging, as discussed by Kaur and Secord [276] in the context of developmental immunology. Persistent NF-κB/MAPK activation also suppresses pro-resolving pathways, including the synthesis of lipoxins, resolvins and protectins, while oxidative stress disrupts mitochondrial function and stem cell-driven epithelial regeneration, thereby delaying tissue recovery. As Taguchi and Mukai [278] have noted, dysregulated membrane trafficking may further prolong receptor signaling, contributing to the chronicity of microplastic-induced inflammation. This results in a prolonged and dysregulated inflammatory response that is more likely to transition into chronic, low-grade inflammation, with long-term consequences for immune development and intestinal homeostasis.

Microplastic-induced inflammation is further intensified by ROS generation, which acts as both an upstream activator and a downstream amplifier of innate immune signaling. Excessive ROS production has been repeatedly demonstrated in cells exposed to nanoparticles, particularly in macrophages and epithelial tissues. For instance, Chen et al. [282] found that polystyrene nanoparticles trigger the robust, ROS-dependent activation of the MAPK and NF-κB signaling pathways. Similarly, Tang et al. [283] demonstrated that MNPLs exacerbate LPS-induced inflammation via the ROS/MAPK axis. These findings highlight ROS as a critical convergence point linking contaminant-driven oxidative stress with receptor-mediated inflammatory activation.

A major enzymatic source of ROS during MNPLs exposure is NADPH oxidase 2 (NOX2). The persistent activation of TLRs 2 and 4, as described by Cui et al. [284] and Antunes et al. [285], promotes NOX2 assembly at the plasma membrane, resulting in sustained superoxide generation. NOX2-derived ROS increase NF-κB and MAPK signaling, while also destabilizing mitochondrial membranes. This creates a feed-forward loop of oxidative stress and cytokine production. This persistent ROS amplification further enhances epithelial barrier dysfunction and promotes inflammatory cell recruitment.

Mitochondrial ROS production and lysosomal destabilization are also central triggers of NLRP3 inflammasome activation. The priming role of PRRs in inflammasome assembly, as described by Li and Wu [274], provides a solid basis for understanding how MNPLs initiate IL-1β maturation. Studies by Tang et al. [283] and Chen et al. [282], for example, support this concept by demonstrating that MNPLs induce mitochondrial dysfunction and ionic imbalance—the canonical signals required for NLRP3 activation. Once activated, the NLRP3 inflammasome promotes the caspase-1-dependent cleavage of pro-IL-1β and pro-IL-18, thereby reinforcing inflammatory signaling and inducing pyroptotic cell death. Pyroptosis-associated membrane rupture may also release additional DA MNPLs, perpetuating PRR activation and inflammatory amplification.

The importance of impaired inflammation resolution during the early stages of development becomes particularly evident when considering the immaturity of PRR-regulated pathways, as described by Li and Wu [274]. They emphasized that neonatal tissues possess underdeveloped regulatory mechanisms required to terminate PRR-driven inflammation. The developing gut, for instance, lacks fully mature programmes for resolving inflammation involving lipid mediators, efferocytosis and epithelial restitution. This makes it particularly susceptible to prolonged inflammatory states triggered by MNPLs. This developmental vulnerability is consistent with the broader insights into developmental immunology provided by Kaur and Secord [276], who noted that immature tissues have a reduced ability to terminate inflammatory signaling. Persistent activation of NF-κB and MAPKs suppresses these resolution pathways, prolonging cytokine production and preventing the transition from inflammation to tissue repair. This observation is consistent with the TLR-driven signaling dynamics described by Takeda and Akira [277], as well as the membrane trafficking-dependent prolongation of signaling highlighted by Taguchi and Mukai [278]. Simultaneously, oxidative stress disrupts mitochondrial function and stem cell-driven epithelial renewal, thereby further delaying tissue recovery. This mechanism is supported by ROS-dependent inflammatory amplification, as demonstrated by Chen et al. [282] and Tang et al. [283]. Consequently, the inflammatory response becomes prolonged and dysregulated, making it more likely to evolve into chronic low-grade inflammation with long-term consequences for immune development and gut homeostasis.

Furthermore, MNPLs trigger inflammation by disrupting the epithelial barrier and activating TLR2, TLR4 and TLR9 signaling simultaneously. This is consistent with the PRR recognition principles outlined by Li and Wu [274], as well as the TLR-specific inflammatory responses demonstrated by Cui et al. [284] for TLR2 and Antunes et al. [285] for TLR4. This dual stimulation results in the persistent activation of the NF-κB and MAPK pathways, including the ERK, JNK and p38 signaling cascades. This process drives the strong induction of IL-1β, IL-6 and TNF-α. These findings are in line with the TLR–NF-κB signaling mechanisms described by Troutman et al. [280] and the PI3K-dependent regulatory constraints identified by Ni et al. [281]. Cytokine production is further amplified by NLRP3 inflammasome activation, whose priming and activation mechanisms correspond closely with the PRR-inflammasome interactions detailed by Li and Wu [274]. The resulting cytokines reinforce oxidative stress through NOX-dependent ROS generation and mitochondrial dysfunction, forming a self-sustaining oxidative-inflammatory loop similar to the ROS-driven amplification observed by Chen et al. [282] and Tang et al. [283]. Such persistent inflammatory activation is particularly detrimental in developing tissues, where epithelial regeneration and antioxidant defenses are not fully established.

Endoplasmic reticulum (ER) stress is an additional factor contributing to microplastic-induced toxicity. Persistent ROS accumulation and cytokine signaling disrupt protein-folding homeostasis, thereby activating the unfolded protein response (UPR). Although ER stress is not traditionally emphasized in classical PRR literature, insights into membrane trafficking provided by Taguchi and Mukai [278] help to explain how disrupted vesicular transport during inflammation can overload the ER with misfolded proteins. UPR activation enhances NF-κB signaling further and sensitizes cells to NLRP3 inflammasome activation, creating a tightly interconnected network of oxidative, inflammatory, and stress-response pathways. Furthermore, prolonged ER stress can promote apoptosis and further compromise the integrity of the epithelial barrier, thereby exacerbating tissue injury [286].

The mechanisms of ROS overproduction, NOX2 activation, NLRP3 inflammasome assembly and ER stress may interact with the TLR-mediated NF-κB/MAPK signaling pathways described by Troutman et al. [280] and Ni et al. [281]. In immature tissues, where antioxidant defenses and ER quality-control systems are underdeveloped, these pathways can remain active for extended periods, leading to increased susceptibility to chronic and dysregulated inflammation. This prolonged signaling environment favors persistent epithelial injury, altered immune maturation and long-term disruption of intestinal homeostasis.

As noted by Kaur and Secord [276], in immature tissues where tight-junction integrity, antioxidant capacity and pro-resolving pathways remain incompletely developed, this inflammatory loop cannot be effectively terminated. The additional burden of ER stress, which is linked to disrupted membrane trafficking and unresolved inflammatory signaling, as described by Taguchi and Mukai [278], further prolongs NF-κB/MAPK activation and sensitizes cells to NLRP3 activation. Consequently, exposure to MNPLs results in prolonged, dysregulated inflammation and increased susceptibility to long-term intestinal injury. The current evidence indicates that exposure to MNPLs and their associated contaminants creates a chronic inflammatory environment in the developing gut, where oxidative stress, PRR activation, and impaired resolution mechanisms converge to cause persistent epithelial dysfunction and immune system malfunction.

## 8. Endocrine Disruption and Developmental Consequences of Microplastic-Associated Contaminants

One of the key mechanisms by which MNPLs -associated contaminants induce developmental toxicity is interference with nuclear hormone receptors. MNPLs act as carriers for endocrine-active chemicals, such as bisphenols, phthalates, PFAS, pesticides and adsorbed metals. These chemicals can bind directly to oestrogen receptors (ERα/β), androgen receptors (AR), peroxisome proliferator-activated receptors (PPARα/γ/δ) and thyroid hormone receptors (TRα/β) [30]. This interaction can alter receptor conformation, co-activator recruitment and DNA-binding affinity, thereby disrupting the transcriptional regulation of hormone-responsive genes [7,33].

As nuclear receptors orchestrate organogenesis, metabolic programming, neurodevelopment and sexual differentiation, the inappropriate activation or suppression of these pathways during critical developmental periods can redirect developmental trajectories permanently. MNPLs intensify these effects by increasing the bioavailability, persistence and tissue accumulation of endocrine-active contaminants, particularly within hormone-sensitive organs and developing endocrine tissues [8,9]. Consequently, MNPLs act not only as passive carriers of pollutants, but also as active modulators of endocrine signaling networks.

Beyond receptor-level interactions, microplastic-associated contaminants interfere with both steroidogenesis and thyroid hormone signaling. For example, phthalates and PFAS inhibit key steroidogenic enzymes such as CYP11A1, CYP17A1 and 3β-hydroxysteroid dehydrogenase (3β-HSD), thereby reducing testosterone, oestradiol and progesterone synthesis [13]. Meanwhile, MNPLs impair the hypothalamic-pituitary-thyroid (HPT) axis by inhibiting thyroid peroxidase (TPO), altering deiodinase activity, and displacing T3/T4 from transport proteins. This ultimately reduces thyroid hormone bioavailability [28,287]. These disruptions are particularly significant during the early stages of life, as thyroid hormones play a crucial role in regulating neuronal differentiation, synaptogenesis, growth, and metabolic maturation. Consequently, disturbances in thyroid signaling during these critical periods of development may result in long-lasting neurological, metabolic and physiological abnormalities [288].

Importantly, MNPLs also interfere with hormone transport and metabolic clearance mechanisms. PFAS, bisphenols and phthalates competitively bind to transport proteins such as transthyretin (TTR) and albumin, thereby displacing endogenous thyroid hormones and reducing their delivery to target tissues [32]. Meanwhile, microplastic-associated contaminants modulate phase I and phase II metabolic enzymes, including cytochrome P_450_ enzymes (CYPs), UDP-glucuronosyltransferases (UGTs) and sulfotransferases (SULTs). This accelerates the degradation of some hormones while impairing the clearance of others [289,290]. This dysregulation disrupts endocrine homeostasis during critical stages of tissue differentiation, neuronal maturation, and metabolic set-point establishment. Consequently, even subtle hormonal imbalances during early development can result in significant long-term physiological consequences.

Although many molecular targets of endocrine disruption have been identified, the precise mechanisms by which exposure to MNPLs leads to altered growth, metabolic programming and maturation of the reproductive axis remain incompletely understood. Nevertheless, there is growing evidence that endocrine disruption induced by MNPLs affects the maturation of the hypothalamic-pituitary-adrenal (HPA), hypothalamic-pituitary-thyroid (HPT) and hypothalamic-pituitary-ovarian/testicular (HPO/HPG) axes. Altered signaling through oestrogen receptors (ERs), androgen receptors (ARs), peroxisome proliferator-activated receptors (PPARs) and thyroid receptors (TRs) within the hypothalamus, pituitary gland and gonads disrupts neuroendocrine programming. This leads to impaired glucocorticoid signaling, altered stress responsiveness and dysregulated energy balance [10,32]. Disruptions to the HPO axis, such as reduced gonadotropin release, altered steroidogenesis and impaired hormonal feedback loops, can delay or hasten puberty, impair gametogenesis and diminish reproductive capacity [27,46]. Importantly, endocrine dysregulation during early development can permanently alter physiological set points, increasing susceptibility to metabolic and reproductive disorders in adulthood.

A critical yet frequently overlooked mechanism underlying these developmental effects is the epigenetic reprogramming induced by endocrine-active contaminants. MNPLs and their associated chemicals can modify DNA methylation patterns, alter histone acetylation and methylation states, and disrupt microRNA expression in hormone-responsive tissues [13,29]. These epigenetic alterations may activate or silence genes involved in metabolism, reproduction, neurodevelopment, and stress regulation. As epigenetic modifications can persist long after exposure has ceased, they provide a biological explanation for the delayed and long-term consequences of early-life endocrine disruption. Furthermore, endocrine-active contaminants can reprogram the epigenome of germ cells, potentially transmitting altered gene expression profiles to subsequent generations [10,13]. This raises important concerns regarding the transgenerational inheritance of developmental vulnerabilities associated with MNPLs.

Disruption to nuclear receptor signaling, hormone synthesis, transport, metabolic clearance and epigenetic regulation produces broad and long-lasting developmental consequences. Altered endocrine signaling during critical developmental periods can lead to metabolic disorders, impaired fertility, neurodevelopmental abnormalities, altered stress responsiveness, and disrupted pubertal timing in later life [32,287]. Epigenetic modifications within germ cells raise the possibility of transgenerational inheritance whereby physiological vulnerabilities emerge in offspring that were never directly exposed to MNPLs [13]. Thus, MNPLs function not only as acute endocrine disruptors, but also as drivers of persistent and potentially heritable developmental risk.

Beyond their direct endocrine effects, MNPLs have broader systemic impacts that increase cellular vulnerability to environmental toxicants. Evidence suggests that long-term exposure to MNPLs interferes with endocrine, metabolic, and developmental pathways, thereby lowering the tolerance threshold for oxidative injury in multiple organ systems [260]. In vascular and endothelial contexts, MNPLs similar in size to polypropylene particles derived from medical injection systems impair cell viability and induce cellular stress responses. This demonstrates that even clinically relevant exposure routes can contribute to a cumulative toxic burden [262]. These findings suggest that the toxicity associated with MNPLs extends far beyond the gastrointestinal tract and may involve systemic endocrine and metabolic dysregulation.

MNPLs can also alter the communication between the gut and the liver, as well as the communication between the gut and the immune system, by reshaping microbial communities and modifying host metabolic signaling pathways. This process promotes systemic inflammation and redox imbalance [262]. In sensory systems, polystyrene MNPLs can penetrate cochlear and neurosensory tissues, inducing mitochondrial dysfunction, ER stress, and apoptotic signaling. This highlights the vulnerability of cell populations that are highly dependent on energy [257]. These observations are consistent with the broader toxicological framework, indicating that micro- and nanoplastics act as xenobiotic stressors that may interact with conventional chemical pollutants in ways that modify carcinogenic, metabolic and immunotoxic pathways [240]. Such multi-organ interactions further complicate the biological consequences of endocrine disruption during development.

Furthermore, MNPLs have been detected in drinking water sources, and emerging organoid-based models demonstrate that these particles can compromise epithelial barrier integrity and alter tissue responses to co-occurring contaminants. This emphasizes their relevance for public health risk assessment [248]. Early-life exposure may be particularly harmful, as activation of the aryl hydrocarbon receptor (AhR) during prenatal chemical exposure can reprogram cardiovascular, renal, and metabolic pathways. This can potentially intensify the long-term consequences of developmental microplastic exposure [247]. These observations are consistent with the broader toxicological framework, indicating that MNPLs act as xenobiotic stressors that may interact with conventional chemical pollutants in ways that modify carcinogenic, metabolic and immunotoxic pathways [240]. Such multi-organ interactions further complicate the biological consequences of endocrine disruption during development.

Thus, current evidence demonstrates that MNPLs -associated contaminants disrupt endocrine function through multiple converging mechanisms, which are particularly detrimental during the early stages of development. By acting as agonists or antagonists of nuclear hormone receptors (ERα/β, AR, PPARs and TRs), these contaminants alter the transcriptional programmes that control organogenesis, metabolic programming and sexual differentiation. They also impair steroidogenesis and thyroid hormone signaling by inhibiting key biosynthetic enzymes, altering deiodinase activity and interfering with TPO function. This reduces the availability of essential sex and thyroid hormones. MNPLs also interfere with the transport and metabolic clearance of hormones by displacing them from carrier proteins and altering the metabolism mediated by CYPs, UGTs and SULTs, ultimately disrupting systemic endocrine homeostasis during sensitive developmental periods [32,287].

These converging disruptions impair the maturation of the HPA, HPT and HPO axes, thereby affecting growth, neurodevelopment, metabolic programming and reproductive capacity. Many endocrine-active contaminants associated with MNPLs also induce epigenetic reprogramming through the modification of DNA methylation, histone marks and microRNA expression in hormone-responsive tissues and germ cells. As these epigenetic changes can persist throughout life and potentially across generations, endocrine disruption caused by MNPLs poses an immediate toxicological threat and a long-term developmental risk. Consequently, MNPLs should be regarded not only as environmental pollutants, but also as biologically active agents that can influence endocrine and developmental processes throughout life [291,292]. Table 4 summarizes the target endocrine axis, contaminant class, model system, developmental stage, measured endpoint and strength of evidence to clarify these distinctions.

Endocrine outcomes are often derived from non-developmental models or high-dose exposures, which limit their applicability to early-life physiology.

Our current understanding of MNPLs as vectors during early development is limited due to several methodological and conceptual gaps. Most existing studies use simplified exposure systems that do not accurately reflect the chemical complexity of environmental mixtures or the changing conditions of neonatal gastrointestinal fluids. Furthermore, experimental studies often use pristine, monodisperse particles, whereas aged plastics in the environment exhibit altered surface chemistry, biofilm formation, and enhanced sorption capacity, all of which may substantially modify biological responses. Another major limitation is the scarcity of developmental models that can capture the full spectrum of early-life physiology, including immature detoxification pathways, endocrine sensitivity, and evolving immune function. Additionally, laboratory dose ranges often exceed environmentally relevant concentrations, making extrapolation to realistic exposure scenarios difficult. These limitations hinder the accurate assessment of long-term endocrine and developmental risks associated with environmentally relevant microplastic exposure.

Therefore, future research should prioritize integrated experimental models that combine environmentally realistic microplastic–contaminant mixtures with developmental stages accurately reflecting neonatal and early-life physiology. High-resolution analytical approaches are essential for characterizing sorption dynamics, desorption kinetics, and particle transformation within immature gastrointestinal environments. Investigations should particularly focus on how particle-associated contaminants modulate oxidative stress pathways, inflammatory signaling networks, and hormone-regulated developmental processes. Longitudinal and multigenerational study designs are crucial for determining whether early-life exposure produces persistent or transgenerational effects. Including diverse polymer types, weathering states and biological matrices in research will also improve current risk assessment frameworks. Ultimately, advancing this field will require the close integration of polymer chemistry, environmental toxicology, endocrinology, and developmental biology to fully understand how MNPLs function as active developmental vectors during critical growth and maturation periods.

## 9. Conclusions

Micro- and nanoplastic particles (MNPLs) can interact with environmental contaminants, modifying exposure profiles during early development. Current evidence indicates that these interactions may influence oxidative balance, inflammatory signaling, epithelial barrier stability and endocrine function, although the strength of these effects varies across models and exposure conditions. Early developmental stages appear to be particularly sensitive to these effects due to immature epithelial barriers, evolving immune networks, limited detoxification capacity and heightened endocrine responsiveness.

Findings from experimental studies suggest that MNPLs and associated chemicals can contribute to redox imbalance, mitochondrial stress, cytokine activation and altered hormone-related pathways. However, many data derive from high-dose exposures, pristine particles or non-developmental models, which limits the ability to infer the magnitude of effects under environmentally relevant conditions. Interactions between mixtures and metals, pesticides, PFAS, endocrine-active compounds and microbial biofilms further complicate interpretation, as their combined influence cannot be predicted from single-compound studies.

Overall, the available research suggests that MNPLs may act as dynamic carriers capable of modulating biological responses during critical developmental periods. Nevertheless, substantial uncertainties remain regarding real-world exposure levels, particle ageing, mixture complexity and long-term outcomes. Addressing these gaps will be essential for accurately assessing the developmental risks associated with MNPLs.

## Figures and Tables

**Table 1:** Comparative overview of physicochemical properties, contaminant sorption mechanisms, degradation behaviors and early-life toxicological outcomes associated with major microplastic polymer types across aquatic and terrestrial model systems.

Polymers	Key Physicochemical Properties	Sorption of Contaminants	Degradation and Weathering	Early-Life Toxicological Outcomes (Model Systems and Principal Findings)	References
Polystyrene [PS]	Aromatic polymer; strong π–π interactions; hydrophobic; readily forms micro- and nanoscale fragments with high cellular penetration potential; surface becomes highly reactive after oxidation	High affinity for hydrophobic organic pollutants; strong π–π binding with aromatic compounds; sorption enhanced by environmental ageing	Rapid UV-induced fragmentation into nanoplastics; oxidation introduces reactive oxygen-containing groups; release of additives (DEHP, DEHT, BPA, BPS) depending on conditions	Models: *Mytilus galloprovincialis*, zebrafish, marine invertebrates. Findings: immune suppression, oxidative stress, apoptosis, reproductive toxicity, locomotor impairment, transcriptomic dysregulation, synergistic toxicity with Cd	[57,58,59,60,67,70,74,78,79,80]
Polyethylene [PE]	Chemically inert; highly hydrophobic; low density; semi-crystalline; most abundant polymer in aquatic systems; limited intrinsic reactivity	Adsorbs lipophilic pollutants; moderate PFAS affinity; sorption increases after oxidation; strong biocorona formation after ageing	Oxidative weathering introduces carbonyl groups; microbial degradation by bacteria and fungi; surface cracking and fragmentation; formation of secondary nanoplastics	Models: *Mytilus galloprovincialis*, zebrafish larvae. Findings: reduced filtration rate, oxidative stress, altered antioxidant enzyme activity, intestinal injury, developmental abnormalities	[6,57,62,63,64,70,74,81]
Polypropylene [PP]	Hydrophobic; low density; high crystallinity; widely used in packaging; environmentally abundant	Lower sorption capacity than PS and PE in pristine form; increases after oxidation; binds hydrophobic pollutants on aged surfaces	UV- and thermal-induced surface oxidation; microbial degradation more pronounced than PE; cracking and generation of reactive surface groups	Models: zebrafish, aquatic larvae. Findings: oxidative stress, ROS induction, digestive enzyme disruption, increased toxicity after weathering	[6,57,61,62,63,64,70,71,72,73,74]
Polyvinyl chloride [PVC]	Chlorine-rich polymer backbone; high density; rigid structure; high content of plasticisers (phthalates); strong additive load	Strong binding of metals (Cd, Pb) and polar contaminants; high affinity for endocrine-disrupting compounds	Releases plasticisers under environmental and physiological conditions; UV-induced fragmentation; formation of reactive oxygen-containing surface groups	Models: *Mytilus galloprovincialis*, zebrafish. Findings: endocrine disruption, oxidative stress, apoptosis, metabolic pathway alteration, synergistic toxicity with Cd, behavioral abnormalities	[57,58,67,68,70,74]
Polyethylene terephthalate [PET]	Aromatic polyester; relatively polar; high density; stable ester bonds; widely used in food and infant exposure contexts	Adsorbs PFAS, pharmaceuticals and metal ions via hydrogen bonding and electrostatic interactions	Resistant to fragmentation; undergoes autocatalytic hydrolysis; releases monomers and oligomers influenced by pH and microenvironment	Models: *Drosophila melanogaster*, zebrafish, *Mytilus galloprovincialis*. Findings: locomotor deficits, neuromuscular dysfunction, developmental delay, sex-specific lifespan effects, metabolic disruption	[57,58,67,70,72,73,74]

Abbreviations: PS—polystyrene; PE—polyethylene; PP—polypropylene; PVC—polyvinyl chloride; PET—polyethylene terephthalate; DEHP—di(2-ethylhexyl) phthalate; DEHT—di(2-ethylhexyl) terephthalate; BPA—bisphenol A; BPS—bisphenol S; Cd—cadmium; Pb—lead; PFAS—per- and polyfluoroalkyl substances; ROS—reactive oxygen species; *Mytilus galloprovincialis*—Mediterranean mussel; *Drosophila melanogaster*—fruit fly.

**Table 2:** Summary of the impacts of micro- and nanoplastics (MNPLs) on fish development, including co-exposure mechanisms and transgenerational outcomes.

Exposure Scenario	Model System	Exposure Concentrations, Durations	Developmental and Physiological Effects	Key Pathways	References
MNPLs (44 nm)	*Danio rerio* embryos/larvae	1, 10, 100 μg/L, 30-day exposure (parental), F1 assessed at 2 hpf	Growth inhibition, reduced hatching, impaired F1 development, neurobehavioral deficits	Disruption of gut–brain–microbiota axis; neurotransmitter imbalance; intestinal inflammation; oxidative stress	[166]
Fluorescent MNPLs uptake	*Danio rerio* embryos/larvae	0.1, 1, 10 ppm, exposure 6–120 hpf, depuration 120–168 hpf	Decreased heart rate; altered swimming behavior; tissue accumulation	Cellular uptake; systemic distribution; organ-level bioaccumulation	[165]
MNPLs + 4-nonylphenol (4-NP)	*Danio rerio*	PS-NPs: 1, 10, 100 μg/L + 4-NP (10 μg/L), chronic 45-day exposure	Neurotoxicity; oxidative stress; neurotransmission disruption; neuronal cell loss	Reduced CAT and GSH; acetylcholinesterase (AChE) inhibition; altered GS/GDH; energy metabolism disruption	[169]
MNPLs + BDE-47	*Hexagrammos otakii* embryos	MNPLs (size not specified) + BDE-47 (concentration not specified in excerpt); exposure during embryonic development; effects measured at hatching stage	Reduced hatchability; mortality; developmental malformations	Wnt signaling activation; xenobiotic metabolism interference; enhanced contaminant uptake	[170]
MNPLs + additives (PS + BPS + MEHP)	*Danio rerio* embryos	PS, BPS, MEHP at non-toxic concentrations, including ≤EC10 for BPS and MEHP; exposure during embryonic development	Severe synergistic malformations	Oxidative stress; thyroid axis disruption (HPT); p53 and CYP1A1 activation	[171]
MNPLs + BDE-47	*Danio rerio* embryos	PS-NPs: concentration not specified + BDE-47, exposure up to 120 hpf; PS-NP aggregation at 12 and 48 hpf; 7-day depuration phase	Edema; spinal curvature; reduced survival	Increased PBDE uptake; endocrine disruption (thyroid axis)	[133]
MNPLs + diclofenac	*Danio rerio* embryos and adults	PS-NPs: 100 μg/L + DCF: 50 or 500 μg/L, 6-day exposure (embryos); adults also exposed (duration not specified)	Mortality; malformations; intestinal damage	Oxidative stress; apoptosis; inflammatory signaling	[172]
MNPLs + BDE-209	*Chlamys farreri* (bivalve)	PS MNPLs: 125 μg/L + BDE-209: 10 or 100 μg/L, exposure duration not specified in excerpt	Cytotoxicity; tissue damage	Enhanced uptake of PBDE; cellular stress responses	[173]
MNPLs (review)	*Danio rerio* embryos	Review article	Neurotoxicity; immunotoxicity; cardiac and gastrointestinal dysfunction	ROS imbalance; apoptosis; microbiota dysbiosis; multi-omics pathway disruption	[164]

Abbreviations: MNPLs—micro- and nanoplastics; PS—polystyrene; PS MNPLs—polystyrene nanoplastics; BPS—bisphenol S; MEHP—mono-(2-ethylhexyl) phthalate; 4-NP—4-nonylphenol; PBDE—polybrominated diphenyl ether; BDE-47—2,2′,4,4′-tetrabromodiphenyl ether; BDE-209—decabromodiphenyl ether; DCF—diclofenac; ROS—reactive oxygen species; AChE—acetylcholinesterase; CAT—catalase; GSH—reduced glutathione; GS—glutamine synthetase; GDH—glutamate dehydrogenase; HPT axis—hypothalamic–pituitary–thyroid axis; p53—tumor suppressor protein p53; CYP1A1—cytochrome P450 1A1.

**Table 3:** Signaling pathways involved in MNPLs -induced tight junction and mucus barrier disruption in the developing gut.

Models	Type of Exposure (Plastic Related)	Stress Biomarkers and Signaling Pathways	Barrier Alterations (TJ, Mucus, Glycocalyx)	References
Caco-2/HT29-MTX (in vitro)	Polystyrene nanoplastics (20–2000 nm)	↑ROS; HO-1/p38 MAPK/IL-10 activation; STAT1/3 activation in goblet-like cells	↓Claudin-1, ↓Occludin, ↓ZO-1; ↑MUC2 (adaptive response); ↑paracellular permeability	[210]
Mice (chronic exposure)	PS NPs in drinking water (0.1–10 mg/L, 32 weeks)	↑ROS, ↑MDA; ↓SOD, ↓GSH-Px; ↑IL-6, ↑TNF-α, ↑IL-1β; NF-κB–mediated inflammation	Villus erosion, crypt loss, inflammatory infiltration; ↓Claudin-1, ↓Occludin, ↓ZO-1; impaired immune barrier	[202]
Mice (duodenum model)	PS NPs + LPS	Strong ROS induction; NF-κB activation; NLRP3 inflammasome activation	Exacerbated duodenal permeability; TJ destabilization; amplified inflammatory response	[203]
Neonatal enterocytes (glycocalyx model)	Developmental immaturity (increased susceptibility to plastic particles)	Reduced sialylation and fucosylation; altered surface charge	Weakened glycocalyx exclusion zone; increased adhesion and uptake of micro-/nanoplastics	[212]
Colon mucus architecture (human/mouse)	Immature or disease-altered mucus exposed to MNPLs	Altered mucin sulphation; reduced glycan density	Looser MUC2 network; increased microplastic penetration; reduced steric/electrostatic filtering	[208,209]
Marine medaka larvae	Environmentally relevant MNPLs	Immune activation; NF-κB–linked cytokine signaling	Impaired epithelial protection; altered mucus–particle interactions	[213]
Mice (PS MNPLs + high-fat diet)	Polystyrene microspheres + HFD	Microbiota dysbiosis; metabolic inflammation (NF-κB–associated)	Mucus thinning; altered mucin glycosylation; increased luminal particle contact	[214]
Human colon (CRC risk model)	Chronic dietary microplastic exposure	Microbiota-driven mucin degradation; inflammatory signaling (NF-κB–linked)	Disrupted colonic mucus barrier; increased epithelial contact; pro-carcinogenic microenvironment	[211]
Gastric epithelium	PS micro- and nanoplastics	↑ROS; Keap1/Nrf2 pathway activation	Gastric mucosal injury; weakened mucus defense; oxidative barrier damage	[216]
Mice (polyethylene MNPLs)	PE MPs (oral exposure)	↑Oxidative stress; ↑inflammatory cytokines (NF-κB–linked)	TJ disruption; epithelial injury; melatonin-mediated restoration of ZO-1/occludin	[217]
Rat intestine (comparator)	Burn injury ± bFGF	↑MLCK (injury); ↓MLCK (bFGF)	bFGF restores ZO-1, claudin-1, occludin; contrast to plastic-induced TJ loss	[198]
IPEC-J2 cells	BPA (plastic monomer) ± selenium nanoparticles	↑NF-κB; ↑IL-1β, IL-6, IFN-γ, IL-17, TNF-α; ↑ERS (PERK, IRE1α, ATF6)	↓ZO-1, ↓Occludin, ↓Claudin-1; SeNPs restore TJ integrity	199
Mice (gut-liver axis)	PS NPs (oral exposure)	↓SIRT1/AMPK; ↑TLR4/NF-κB; ↑oxidative stress	↓ZO-1, ↓Occludin, ↓Claudin-1; mucus and microbiota disruption; liver injury secondary to barrier failure	[207]
Biofilm-coated MNPLs	MNPLs with microbial biofilms + heavy metals	Metal dysregulation; ↑ROS; mitochondrial stress (NF-κB–linked)	Enhanced adhesion to mucus; increased epithelial exposure; potential neurotoxic metal delivery	[172]

Abbreviations: ↑—increase/upregulation; ↓—decrease/downregulation; AMPK—AMP-activated protein kinase; ATF6—activating transcription factor 6; BPA—bisphenol A; bFGF—basic fibroblast growth factor; CRC—colorectal cancer; ERS—endoplasmic reticulum stress; GSH-Px—glutathione peroxidase; HFD—high-fat diet; HO-1—heme oxygenase-1; IFN-γ—interferon gamma; IL—interleukin; IRE1α—inositol-requiring enzyme 1 alpha; Keap1—Kelch-like ECH-associated protein 1; LPS—lipopolysaccharide; MAPK—mitogen-activated protein kinase; MDA—malondialdehyde; MLCK—myosin light-chain kinase; MUC2—mucin-2; NF-κB—nuclear factor kappa-light-chain-enhancer of activated B cells; NLRP3—NOD-like receptor family pyrin domain-containing 3 inflammasome; NPs—nanoplastics; PE MNPLs—polyethylene MNPLs; PERK—protein kinase RNA-like endoplasmic reticulum kinase; ROS—reactive oxygen species; SeNPs—selenium nanoparticles; SIRT1—sirtuin 1; SOD—superoxide dismutase; STAT1/3—signal transducer and activator of transcription 1/3; TJ—tight junction; TLR4—Toll-like receptor 4; TNF-α—tumour necrosis factor alpha; ZO-1—zonula occludens-1.

**Table 4:** Summary of endocrine-related effects of microplastics and associated contaminants across axes, model systems, developmental stages and strength of evidence.

Endocrine Axis	Contaminant Class	Model System	Developmental Stage	Endpoint Measured	Strength of Evidence	Reference
HPA axis	PFAS	Human cell lines; rodent models	Fetal/neonatal	Cortisol signaling, GR expression, stress-axis activation	Moderate (animal + in vitro)	[3,18,19,20,21,22,23]
HPA axis	MNPLs particles	Zebrafish; rodent juveniles	Early development	Cortisol dysregulation, altered stress reactivity	Moderate (animal)	[6,23,57,62,63,64,70,74,81]
HPT axis	Sorbed EDCs (BPA, phthalates)	Human thyroid cell lines; zebrafish embryos	Embryonic/larval	T3/T4 disruption, altered deiodinase activity	Strong (multiple models)	[28,287,288]
HPT axis	MNPLs additives	Rodent pups	Postnatal	Thyroid hormone synthesis and receptor expression	Moderate	[32,287]
HPG axis	PFAS	Rodent juveniles; human granulosa cells	Pre-pubertal	Steroidogenesis, gonadal development, hormone receptor expression	Strong (consistent across models)	[245,247,248]
HPG axis	MNPLs + additives	Zebrafish; rodent neonates	Early development	Sex hormone imbalance, delayed gonadal maturation	Moderate	[6,23,57,61,62,63,64,70,71,72,73,74]
GH/IGF axis	MNPLs	Zebrafish larvae	Early development	Reduced growth rate, altered IGF expression	Moderate	[6,57,61,62,63,64,70,71,72,73,74]
GH/IGF axis	Metals (e.g., Cd, Pb)	Rodent pups	Postnatal	Growth suppression, pituitary signaling	Strong	[57,58,67,68,70,74]
Pancreatic axis	Sorbed EDCs	Human β-cell lines; rodent neonates	Early life	Insulin secretion, β-cell stress	Strong	[28,287,288]
Pancreatic axis	Microplastics	Zebrafish larvae	Early development	Glucose dysregulation, oxidative stress	Emerging	[57,58,59,60,67,70,74,78,79,80]
Cross-axis effects	Secondary inflammation	Rodent neonates; intestinal organoids	Early development	Cytokine-driven endocrine disruption	Moderate	[165,166,169]
Cross-axis effects	Mixed exposures (MNPLs + PFAS/EDCs)	Human cell lines; zebrafish	Embryonic/larval	Hormone receptor signaling, oxidative stress, epigenetic marks	Emerging to moderate	[3,18,19,20,21,22,23]

Abbreviations: HPA—hypothalamic–pituitary–adrenal axis; HPT—hypothalamic–pituitary–thyroid axis; HPG—hypothalamic–pituitary–gonadal axis; GH/IGF—growth hormone/insulin-like growth factor axis; MNPLs—micro- and nanoplastic particles; EDCs—endocrine-disrupting chemicals; PFAS—per- and polyfluoroalkyl substances; GR—glucocorticoid receptor; T3/T4—triiodothyronine/thyroxine; IGF—insulin-like growth factor.

## Data Availability

No new data were created or analyzed in this study.

## Abbreviations

ABC–ATP-binding cassette; ABCB1 (P-gp)–ATP-binding cassette subfamily B member 1; ABCC2 (MRP-2)–ATP-binding cassette subfamily C member 2; ABCG2 (BCRP)–ATP-binding cassette subfamily G member 2; AChE–acetylcholinesterase; AMPK–AMP-activated protein kinase; ARE–antioxidant response element; ATF6–activating transcription factor 6; ATP–adenosine triphosphate; BDE-47–2,2′,4,4′-tetrabromodiphenyl ether; BDE-209–decabromodiphenyl ether; BPA–bisphenol A; BPS–bisphenol S; bFGF–basic fibroblast growth factor; CAT–catalase; Cd–cadmium; CRC–colorectal cancer; CYP1A1–cytochrome P450 1A1; CYP450–cytochrome P450; DCF–diclofenac; DEHP–di(2-ethylhexyl) phthalate; DEHT–di(2-ethylhexyl) terephthalate; DEP–diethyl phthalate; DRP1–dynamin-related protein 1; EDCs–endocrine-disrupting chemicals; ERS–endoplasmic reticulum stress; ETC–electron transport chain; GDH–glutamate dehydrogenase; GH/IGF–growth hormone/insulin-like growth factor axis; GR–glucocorticoid receptor; GSH–glutathione; GPx–glutathione peroxidase; GST–glutathione S-transferase; GCLC–glutamate–cysteine ligase catalytic subunit; HFD–high-fat diet; HO-1–heme oxygenase-1; HPG–hypothalamic–pituitary–gonadal axis; HPA–hypothalamic–pituitary–adrenal axis; HPT–hypothalamic–pituitary–thyroid axis; IFN-γ–interferon gamma; IGF–insulin-like growth factor; IL–interleukin; IL-1β–interleukin-1 beta; IRE1α–inositol-requiring enzyme 1 alpha; Keap1–Kelch-like ECH-associated protein 1; LPS–lipopolysaccharide; MAPK–mitogen-activated protein kinase; MDA–malondialdehyde; MEHP–mono-(2-ethylhexyl) phthalate; MLCK–myosin light-chain kinase; MNPLs–micro- and nanoplastic particles; MUC2–mucin-2; MyD88–myeloid differentiation primary response protein 88; NF-κB–nuclear factor kappa-light-chain-enhancer of activated B cells; NLRP3–NOD-like receptor family pyrin domain-containing 3; NQO1–NAD(P)H quinone dehydrogenase 1; Nrf2–nuclear factor erythroid 2–related factor 2; NPs–nanoplastics; OPA1–optic atrophy 1; PBDEs–polybrominated diphenyl ethers; PE–polyethylene; PE MNPLs–polyethylene micro- and nanoplastics; PERK–protein kinase RNA-like endoplasmic reticulum kinase; PET–poly(ethylene terephthalate); PFAS–per- and polyfluoroalkyl substances; Pb–lead; PP–polypropylene; PS–polystyrene; PS MNPLs–polystyrene micro- and nanoplastics; PVC–poly(vinyl chloride); ROS–reactive oxygen species; SCFAs–short-chain fatty acids; SeNPs–selenium nanoparticles; SIRT1–sirtuin 1; SOD–superoxide dismutase; STAT1/3–signal transducer and activator of transcription 1/3; St–styrene; TJ–tight junction; TLR4–Toll-like receptor 4; TNF-α–tumour necrosis factor alpha; T3/T4–triiodothyronine/thyroxine; ULK1–unc-51-like autophagy activating kinase 1; Wnt–wingless/integrated signaling pathway; ZO-1–zonula occludens-1.
